# Microenvironment Remodeling Micelles for Alzheimer's Disease Therapy by Early Modulation of Activated Microglia

**DOI:** 10.1002/advs.201801586

**Published:** 2018-12-12

**Authors:** Yifei Lu, Zhongyuan Guo, Yujie Zhang, Chao Li, Yu Zhang, Qin Guo, Qinjun Chen, Xinli Chen, Xi He, Lisha Liu, Chunhui Ruan, Tao Sun, Bin Ji, Weigen Lu, Chen Jiang

**Affiliations:** ^1^ Key Laboratory of Smart Drug Delivery Ministry of Education State Key Laboratory of Medical Neurobiology Research Center on Aging and Medicine Department of Pharmaceutics School of Pharmacy Fudan University Shanghai 201203 China; ^2^ Department of Functional Brain Imaging Research National Institute of Radiological Sciences National Institute for Quantum and Radiological Science and Technology Chiba 263‐8555 Japan; ^3^ National Pharmaceutical Engineering and Research Center China State Institute of Pharmaceutical Industry Shanghai 201203 China

**Keywords:** Alzheimer's disease, early phase, microenvironment modulation, microglia, polymeric micelles

## Abstract

Current strategies for Alzheimer's disease (AD) treatments focus on pathologies in the late stage of the disease progression. Poor clinical outcomes are displayed due to the irreversible damages caused by early microglia abnormality which triggers disease development before identical symptoms emerge. Based on the crosstalk between microglia and brain microenvironment, a reactive oxygen species (ROS)‐responsive polymeric micelle system (Ab‐PEG‐LysB/curcumin (APLB/CUR)) is reported to normalize the oxidative and inflammatory microenvironment and reeducate microglia from an early phase of AD. Through an β‐amyloid (Aβ) transportation‐mimicked pathway, the micelles can accumulate into the diseased regions and exert synergistic effects of polymer‐based ROS scavenging and cargo‐based Aβ inhibition upon microenvironment stimuli. This multitarget strategy exhibits gradual correction of the brain microenvironment, efficient neuroprotection, and microglia modulation, leading to decreased Aβ plaque burdens and consequently enhanced cognitive functions in APPswe/PSEN1dE9 model mice. The results indicate that microglia can be exploited as an early target for AD treatment and their states can be controlled via microenvironment modulation.

## 1 Introduction

Alzheimer's disease (AD) is a common neurodegenerative disease featured by the abnormal cerebral deposition and cognitive dysfunctions.^1^ Current therapeutic agents for clinical or preclinical AD treatment include: 1) cholinergic drugs with the aim of compensating the loss of neurotransmitter, 2) brain‐derived neurotrophic factors (BDNF) to protect damaged neurons, and 3) therapeutic genes to eliminate or inhibit protein aggregation. These strategies perform by achieving temporal neuronal function recovery, neuroprotection, or inhibition of abnormal protein aggregation.^2^ However, researchers have found that diagnostic mild cognitive impairment is the hallmark of an already late stage in AD progression when irreversible brain damage occurs and cannot be cured by neuroprotection or the removal of amyloid deposition.^3^ This could explain the successive failures of Aβ antibodies in clinical trials^4^ during late years. It also calls for the development of new and efficient strategies addressing early pathological changes in AD progression.

Microglia are the intrinsic cerebral immune cells whose number and functions are precisely tailored by brain environment.^5^ Microglia could secret proinflammatory or anti‐inflammatory cytokines in response to pathogens, aberrant proteins or tissue damages; they also function as phagocytes and eliminate cell debris to maintain hemostasis. Normally, microglia activation is controlled with inhibitive neuronal ligands. But under the circumstance of AD, continuous neuron loss causes insufficient inhibition and induces microglia “priming,” a phenotype with hyperreactivity but impaired phagocytosis. Primed microglia are extremely sensitive to immune stimulus which lead to an exaggerated and uncontrolled inflammatory response.^6^ As a result, the abnormal AD microenvironment has a significant impact on the behaviors of primed microglia and accelerates AD progression.^7^ Nanoparticles have been reported effective in controlling microglia states by the modulation of AD microenvironment, mainly through the removal of toxic proteins. For example, high‐density lipoprotein/apolipoprotein E nanoparticles enhanced the clearance of β‐amyloids and alleviated microglia overactivation;[[qv: 8a]] brain‐targeted nanoparticles also inhibited neuroinflammation by disrupting Aβ aggregation via siRNA or therapeutic peptides delivery;[[qv: 2b,8b]] ceria‐based (CeO_2_) nanoparticles reduced tau‐induced microglial activation by delivering tau inhibitor.[[qv: 8c]] However, clinical data show that anti‐inflammatory strategies only function when applied in an early, asymptomatic phase of AD.^7^ Although abnormal protein aggregation and neuron damage are used to define AD pathology, they are considered as rather the result than the pathogeny of disease progression. Current strategies are mostly based on pathologies in the late, irreversible stage of AD, proven to be ineffective in clinical trials.^9^

It is reported that cellular stress and synapse loss should occur years before the emergence of amyloid deposition,^10^ while abnormal gliosis starts early and continues throughout the whole AD development.^11^ These two pathologies make a better temporal match and indicate the early involvement of microglia in neuron damage. Oxidative stress caused by high level of reactive oxygen species (ROS) is one of the typical characteristics of AD brains.^12^ ROS could be generated early from activated microglia and damaged mitochondria in affected neurons, or from the reaction between metal ions and Aβ amyloids in the advanced stage^13^ that forms a sustaining oxidative microenvironment during AD progression. The gradual accumulation of oxidative damage precedes and leads to the appearance of pathological AD symptoms.^14^ Furthermore, ROS have been reportedly a contributor of microglia activation as proinflammatory signaling molecules.^15^ Similar to the situation in tumor microenvironment, oxidative stress is leveraged to trigger drug release in ROS‐responsive drug delivery systems for AD treatment.^16^ Additionally, in the scenario of AD, these strategies are able to eliminate excessive ROS generated from neuronal mitochondria, to achieve inhibited ROS stimulation to microglia and modulation of the inflammatory microenvironment.[[qv: 8c]]

The hindrance of blood–brain barrier (BBB) and the locus‐specific intracerebral drug distribution should also be taken into consideration for AD drug delivery. Conventional brain‐targeting nanoparticles rely on diffusion once crossing BBB, thus lack AD locus selectivity since only hippocampus and cortex are affected in the early stage.[[qv: 1a]] Notably, substance transportation of BBB is changed under the chronic inflammatory microenvironment induced by abnormal microglia. Downregulation of glucose transporter and low‐density lipoprotein receptor‐related protein has been introduced as neopathy in AD,^17^ which further restricts the application of the already established brain‐targeting strategies. However, expression of receptor for advanced glycation end‐products (RAGE) has been shown to increase on BBB, neurons, and microglia along with the formation of neuroinflammation in AD.^18^ RAGE mediates the influx transport of plasma Aβ into brain and causes subsequent neurotoxicity and microglia activation, which detrimentally contributes to AD pathologies.^19^ Recent work has confirmed RAGE as a solid target for positron emission computed tomography (PET) imaging of AD locus.^20^

**Scheme 1:**
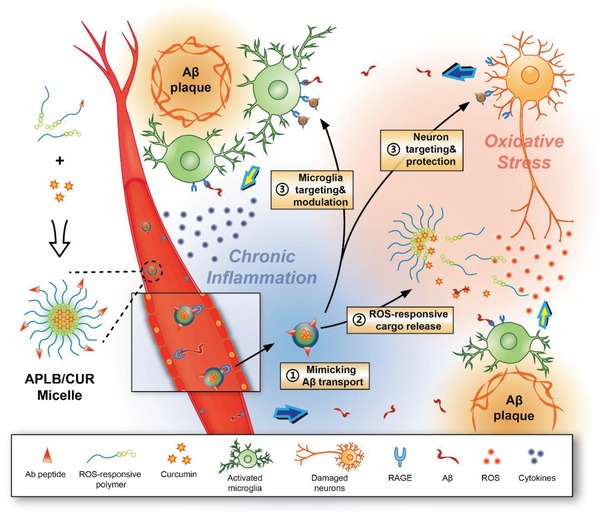
Illustration of microglia induced AD microenvironment and mechanisms of APLB/CUR modulation: 1) Ab peptide modified micelles mimic Aβ transportation from peripheral into brain parenchyma; 2) ROS‐responsive release of ROS scavenging polymer and Aβ inhibitive curcumin; and 3) Aβ‐mimicked micelle targeting to activated microglia and damaged neurons.

Based on the aberrant hyperreactive state of microglia and the associated microenvironment changes in the early stage of AD progression, we herein present a polymeric micelle drug delivery system (Ab‐PEG‐LysB/CUR) with sequential targeting ability to normalize AD microenvironment via microglia modulation. The nanoscale micelles could be constructed with three components, including an RAGE targeting peptide (Ab) derived from Aβ protein, an amphiphilic polymer (poly(ethylene glycol) (PEG)‐LysB) with ROS responsiveness and scavenging ability, and the model drug curcumin (CUR), a hydrophobic natural compound which has been reported to target to Aβ aggregation.^21^ By mimicking abnormal Aβ transportation, the micelles could accumulate in AD microenvironment via RAGE binding and exert neuroprotection and microglia modulation through the synergistic effects of polymers and payloads (**Scheme** **1**). Effective neuroprotection and alleviation of oxidative stress was demonstrated in vitro. It was also proved in amyloid precursor protein (APP)/presenilin‐1 (PS1) mice model that the progressive aggregation of abnormal proteins and the cognitive decline in the late disease stage could be reversed via an early modulation of the oxidative microenvironment and the overreactive state of microglia. This work could provide new evidence for the feasibility to modulate microglia as a new strategy for early phase AD treatment.

## 2 Result and Discussion

### 2.1 Preparation and Characterization of APLB/CUR Micelles

The amphiphilic copolymer was synthesized via amidation reaction between a phenylboronic containing motif and amine groups on poly(ethylene glycol)‐polylysine (PEG‐pLys) (Figure S1, Supporting Information). Briefly, PEG‐pLys was obtained by ring‐opening reaction and the exposed amine side groups were conjugated with an active imidazoyl carbamate of pinacol phenylboronic ester described in a previous work.^22^ The chemical composition of the polymer was verified by ^1^H NMR and gel permeation chromatography (GPC) (Figure S2, Supporting Information) in detail. Approximately 20 phenylboronic groups were attached to each polymer backbone, which formed the hydrophobic segment (designated LysB) to load insoluble curcumin in the inner core via supramolecular self‐assembly. To achieve RAGE‐mediated targeting, a small peptide KLVFFAED (designated Ab peptide) derived from the binding domain of Aβ protein with RAGE was applied.^23^ The C terminal of the peptide was modified with hexynoic acid to introduce an alkynyl group for the click reaction with N_3_‐PEG‐LysB. Successful conjugation was indicated by decreased elution time in GPC result (Figure S2C, Supporting Information) and IR spectrum as the peak of azide group disappeared (Figure S3, Supporting Information).

**Figure 1:**
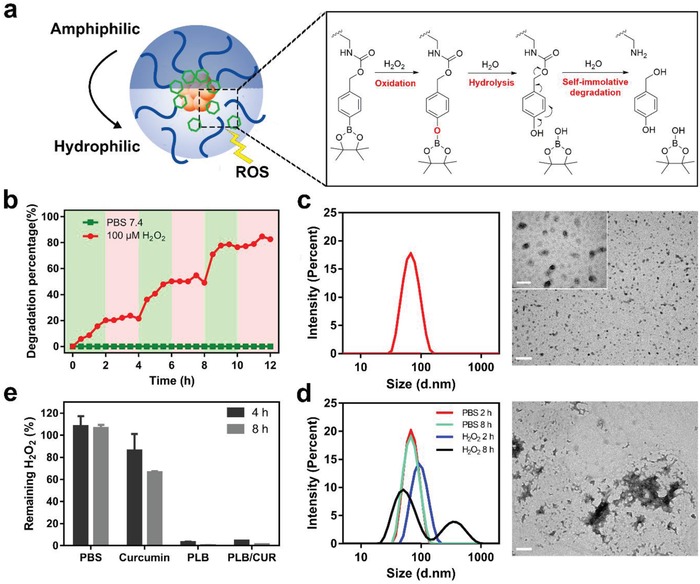
ROS responsiveness of APLB polymer and formulated micelles. a) The mechanism of ROS induced self‐immolative degradation. b) The kinetics of polymer degradation under PBS 7.4 or pulsed H_2_O_2_ (100 × 10^−6^ m); green blocks indicate the presence of H_2_O_2_ stimulation and red blocks indicate no H_2_O_2_ stimulation. c) Size and morphology of APLB/CUR micelles measured by dynamic light scattering (DLS) (left) and TEM (right, scale bar: 200 nm; inset scale bar: 50 nm). d) Size distribution and morphology of APLB/CUR micelles after incubation with 100 × 10^−6^ m H_2_O_2_. e) ROS scavenging ability of micelles.

Due to the responsiveness of phenylboronic structure to oxidative stimuli,^24^ the hydrophobic segment was designed to go through sequential oxidation and hydrolysis as illustrated in **Figure** **1**a. The exposed phenolic hydroxyl group would lead to a cascade of self‐immolative electronic elimination process, which eventually leads to the complete void of the modified motifs. The polymer responsive degradation was examined in vitro with 1 × 10^−6^ m H_2_O_2_ in phosphate buffer saline (PBS) 7.4 to simulate pathological oxidative environment in vivo.^25^ 4‐Hydroxybenzylalcohol was detected as the degradation product from the polymer backbone by high performance liquid chromatography (Figure S4, Supporting Information) and was used to monitor the degradation rate of polymer in H_2_O_2_. As shown in Figure 1b, the polymer demonstrated a pulsatile degradation pattern, which gradually ceased as H_2_O_2_ went out but quickly resumed with additional H_2_O_2_ in each cycle. In consensus, no degradation was found in polymers treated with only PBS.

To prepare the curcumin loaded micelles, a mixture of drug and polymers in *N*,*N*‐dimethylformamide (DMF) was dialyzed against PBS 7.4 overnight to allow self‐assembly. Modification of the RAGE targeting peptide was controlled by quantitative mixing of peptide conjugated polymers. Total three kinds of micelles were prepared: Ab peptide modified or unmodified micelles with curcumin loaded (designated APLB/CUR or PLB/CUR, respectively), and additionally Ab peptide modified micelles without drug loaded (designated APLB). Dynamic light scattering (DLS) and transmission electron microscope (TEM) images showed uniform sphere‐like particles of PLB/CUR with a hydrodynamic diameter around 65 nm (Figure 1c and Figure S5 and Table S1, Supporting Information). The critical micelle concentration of APLB/CUR were measured by using pyrene as a probe to indicate micelle formation and was calculated as 82 µg mL^−1^ (Figure S6, Supporting Information), which was much lower than the micelle concentration in blood (≈500 µg mL^−1^) and guaranteed the stability of micelles in vivo upon dilution. The core–shell structure was readily disassociated after H_2_O_2_ incubation because of the irregular size distribution and micelle morphology (Figure 1d). This was in consistence with the polymer degradation profile and could be attributed to the conversion of the polymer from an amphiphilic state into a more hydrophilic state in respond to ROS. To be mentioned, the ROS responsive drug release profile was not investigated in this work due to the antioxidant property of curcumin which made it hard to detect after incubation with H_2_O_2_.

Next, we evaluated the ROS scavenging ability of micelles as part of their therapeutic effects. H_2_O_2_ was incubated with curcumin or micelles for certain time and its remaining concentration was measured. Both blank micelles (PLB) and curcumin loaded micelles (PLB/CUR) showed excellent ability of ROS scavenging (Figure 1e) compared with free curcumin, as only less than 5% H_2_O_2_ was left after the first 4 h treatment. After further incubation, H_2_O_2_ treated with curcumin also showed slightly decrease which could be attributed to its fewer ROS reaction sites compared with the polymers. This was consistent with the result of a polymeric prodrug of vanillin reported by Kwon et al.^26^ that polymer‐based nanoparticles could provide greater capability to scavenge ROS compared with conventional small molecule antioxidants. Since oxidative stress would cause direct and irreversible neuron death^27^ plus microglia activation,^28^ we suggest that this polymer‐based micelle itself could serve as a strong neuroprotective and anti‐inflammatory agent by inhibiting ROS signaling.

### 2.2 Investigation on Cellular Uptake and Intracellular Disposition of Micelles

KLVFFAED is a short version of Aβ with high binding affinity with RAGE but depleted signaling transduction,^23^ which induces negligible cytotoxicity compared with Aβ and is regarded ideal for micelle functionalization. To substantiate its efficacy, brain capillary endothelium cells (BCECs) and SH‐SY5Y cells were used as the in vitro models for BBB and neurons respectively as both cell lines have been reported to express RAGE.^29^ Cellular uptake was characterized by the fluorescence of curcumin. We found significantly increased fluorescence signal in both cells treated with APLB/CUR compared with unmodified PLB/CUR (Figure S7, Supporting Information), indicating the positive role of Ab peptide in RAGE binding and cell uptake. To further prove that RAGE mediates the transportation of APLB/CUR micelles across BBB, BCECs monolayer in a transwell system was applied as a more similar model to the situation in vivo (Figure S8a, Supporting Information). Coumarin‐6 (Cou6) was encapsulated in the micelles as a probe to detect their permeability across the BCECs monolayer. No significant changes were found in the integrity and stability of the monolayers before and after the experiment (Figure S8b, Supporting Information). Thus, we next investigated if Ab peptide could facilitate micelle transportation across the BCECs monolayer. Coumarin‐6 was detected by high performance liquid chromatography (HPLC) and the permeability of different micelles were presented as apparent permeability coefficient (Papp) and plotted versus time of transport (Figure S8c, Supporting Information). As a result, micelles modified with Ab peptide showed higher transport across the monolayer compared with unmodified micelles and preincubation with free peptide could further inhibit this RAGE‐mediated transport.

**Figure 2:**
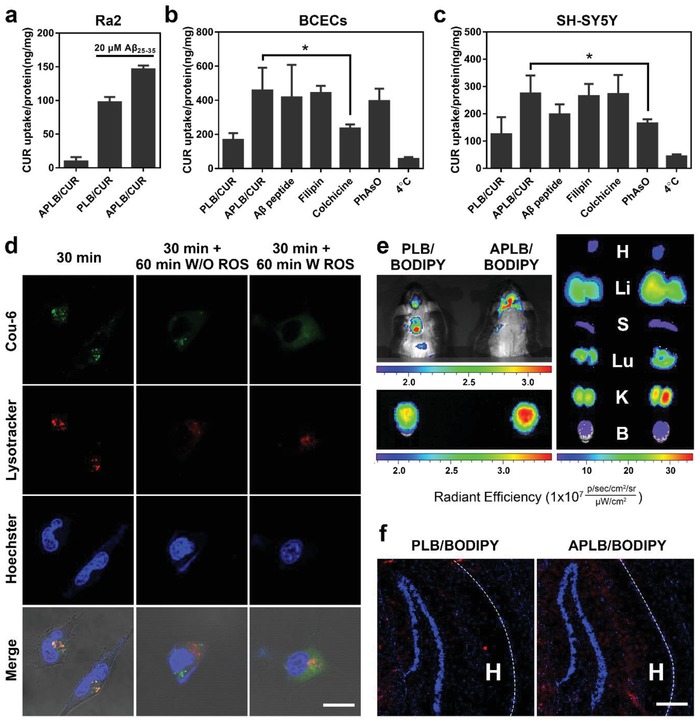
Disposition of APLB/CUR micelles in vitro and in vivo. a) Cellular uptake in Aβ activated Ra2 microglia cells. b,c) Cellular uptake in BCECs and SH‐SY5Y cells under different inhibitive conditions. d) Drug release in SH‐SY5Y cells with or without the incubation of 100 × 10^−6^ m H_2_O_2_ (scale bar: 20 µm). e) In vivo imaging of mice administrated with PEG‐LysB/BODIPY with (right lane) or without (left lane) Aβ modification. Images were taken 4 h after administration. Organs were labeled with the initial character (H for heart, Li for liver, S for spleen, Lu for lung, K for kidney, and B for brain). f) Fluorescence imaging of the brain hippocampus sections after ex vivo in vivo imaging system (IVIS) imaging (red: BODIPY; blue: 4′,6‐diamidino‐2‐phenylindole (DAPI); scale bar: 100 µm). Dashed lines indicate the border of hippocampus (H).

Since RAGE expression can be further upregulated on microglia upon Aβ stimulation,[[qv: 18a]] we investigated the micelle uptake in mouse microglia Ra2 cells incubated with Aβ_25–35_, which is the residue of Aβ but retains the toxicity of the full‐length protein.^30^ Intracellular curcumin quantification demonstrated an over tenfold increase of micelle uptake after 24 h preincubation with Aβ and an even higher uptake of APLB/CUR compared with PLB/CUR due to possible RAGE upregulation and RAGE‐mediated internalization (**Figure** **2**a). We suggested that Ab peptide modified micelles could selectively target to the overreactive subpopulation of microglia to achieve AD microenvironment modulation.

The mechanisms of micelle internalization were further investigated under various inhibitive conditions, including filipin for caveolin inhibition, colchicine for macropinpcytosis inhibition, PhAsO for clathrin inhibition, and 4 °C for energy inhibition. As indicated in Figure 2b,c, the uptake of APLB/CUR was inhibited differently in BCECs and SH‐SY5Y cells, which, respectively, indicated a macropinocytosis pathway and a clathrin‐dependent pathway. This could be attributed to the Ab peptide mimicked different dispositions of Aβ by RAGE on BBB and neurons,^19^ as Aβ crosses BBB from luminal side into brain parenchyma through a transcellular pathway but is eventually internalized by neurons. To verify the drug release profile responding to the intracellular milieu after internalization, micelles were labeled with coumarin and incubated with SH‐SY5Y cells with or without the addition of H_2_O_2_. Micelles were found colocalized with endosomes/lysosomes within the first 30 min and showed effective endosome escaping as separate signals of coumarin (green) and lysosomes (red) were observed after further incubation. However, coumarin could be readily released from the escaped micelles during further incubation with the presence of H_2_O_2_, as the green fluorescence diffused in the whole cytoplasm (Figure 2d), which was consistent with the responsive micelle disassembly observed by TEM. This ensured the micelles to transport through BBB in an intact form via a “Trojan horse” way and then release carried drug in neurons or microglia under oxidative intracellular milieu.

### 2.3 Evaluation of Brain‐Targeting Ability and Intracerebral Distribution

To further validate the Aβ mimicking ability of the micelles, we next studied if the Ab peptide modification could change their biodistribution in vivo. RAGE expression was first examined in the APP/PS1 model to confirm the applicability. Significantly enhanced expression was found in AD affected areas, including cortex and hippocampus, with ≈2.5‐fold increase compared with the wild type mice (Figure S9, Supporting Information). RAGE upregulation enhances the inflammatory milieu around BBB and induces Aβ accumulation in AD brains.^19^ Thus, a hydrophobic near‐infrared probe boron‐dipyrromethene (BODIPY) was encapsulated in the APLB micelles and injected intravenously to APP/PS1 and wild type (WT) mice. More brain accumulation was found in AD mice treated with APLB/BODIPY (Figure 2e and Figure S10, Supporting Information), possibly due to the enhanced RAGE transportation across BBB. BODIPY micelles treated mice were sacrificed and brains were used to perform frozen section. As demonstrated in the fluorescence imaging, more red signals of the probe were found specifically in the hippocampus of mice treated with APLB/BODIPY (Figure 2f) compared with unmodified micelles, indicating the selective accumulation in AD affected areas.

Taken together, this Aβ‐derived peptide mimicked the aberrant Aβ transportation to facilitate BBB penetration and subsequent locus‐specific accumulation of the micelles into AD microenvironment. Then, micelles could be actively internalized into neurons or activated microglia and release cargo in the oxidative intracellular milieu.

### 2.4 In Vitro Evaluation of Neuroprotection Under Oxidative Stress and Aβ Toxicity

**Figure 3:**
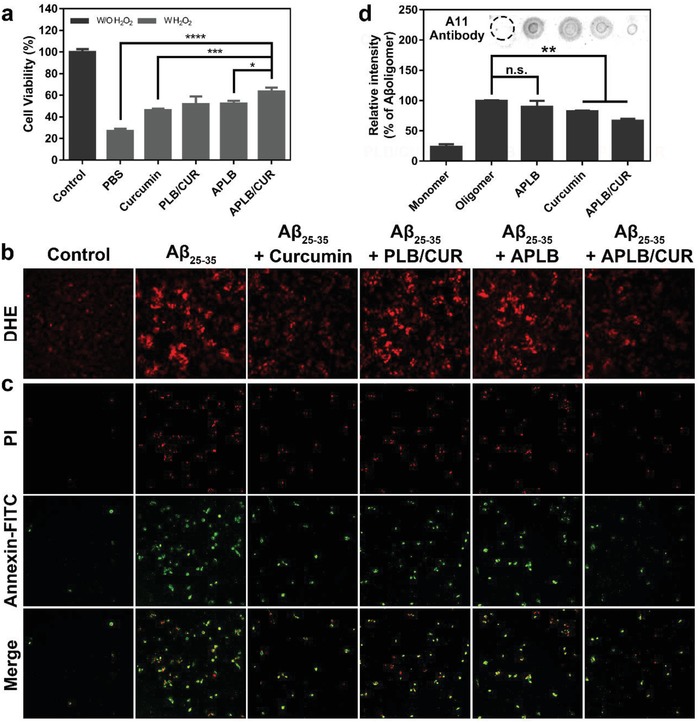
Neuroprotection of APLB/CUR micelles in SH‐SY5Y toxicity cell models. Cells were preincubated with different CUR formulations and then challenged with toxic substances to simulate AD microenvironment. a) Cell viability in the presence of H_2_O_2_ (error bars represent mean ± standard deviation (SD), *n* = 6). b) Cellular oxidative stress in the presence of Aβ_25–35_ (red: oxidized DHE fluorescence, original magnification = 100×). c) Cell apoptosis in the presence of Aβ_25–35_ (green: early apoptosis cells, red: late apoptosis cells, original magnification = 100×). d) Dot blot image of Aβ_25–35_ after incubation with different formulations and quantitative results. *P* value in (a) was calculated by comparing with group APLB/CUR; *P* value in (d) was calculated by comparing with group Aβ oligomer (*P< 0.05, *P< 0.01, ***P< 0.001 or ****P< 0.0001).

To examine the neuroprotective effects of the micelles in AD microenvironment, we applied two neuron toxicity models of SH‐SY5Y via respective incubation with H_2_O_2_ or aggregated Aβ_25–35_ to simulate AD microenvironment.^31^ Previous literatures report that once oxidation occurs in neuronal soma, neurons would go through rapid caspase‐dependent apoptosis.^27^ Therefore, thiazolyl blue (MTT) assay was used to evaluate cell viability in the presence of H_2_O_2_ with preincubation of different formulations. We found severe neuron death after H_2_O_2_ treatment. However, APLB/CUR and APLB significantly rescued cell death compared with PBS, which was attributed to their quick scavenge of large amount of ROS via polymer degradation (**Figure** **3**a). Curcumin also partially contributed to the neuroprotection as drug loaded micelles showed slightly better effect than blank micelles, which agreed with curcumin's ROS scavenging ability as demonstrated in Figure 1e.

Intracellular ROS level could be elevated by Aβ through disruption of mitochondria membrane in neurons, which is one of the mechanisms of Aβ induced neurotoxicity.^32^ Hence, the cellular oxidative stress could be investigated to evaluate the neuroprotective effect of micelles against Aβ after treatment. ROS was directly indicated with an ROS probe dihydroethidium (DHE) in the Aβ_25–35_ treated cell model (Figure 3b). Surprisingly, curcumin treated cells showed least red fluorescence comparable with APLB/CUR group. To further confirm this phenomenon, we also performed Annexin V‐fluorescein (FITC)/propidium iodide (PI) double staining assay and found better antiapoptosis effect of both curcumin and APLB/CUR against Aβ_25–35_ (Figure 3c). The increased efficiency of curcumin in reducing oxidative stress and neuroprotection in this model might attribute to its hydrophobicity facilitated diffusion across cell membrane and its ability to directly bind Aβ oligomers and inhibit their cytotoxicity.^33^ To validate this hypothesis, Aβ monomer was left for aggregation with the presence of different formulations and probed with oligomer‐specific antibody. Curcumin and curcumin loaded micelles showed significant inhibition of Aβ aggregation while negligible effect was shown in blank micelles or PBS treated group, indicating the role of curcumin in Aβ oligomer formation and subsequent neuroprotection (Figure 3d).

### 2.5 Evaluation of Memory Decline, Aβ Burden in AD Model Mice

**Figure 4:**
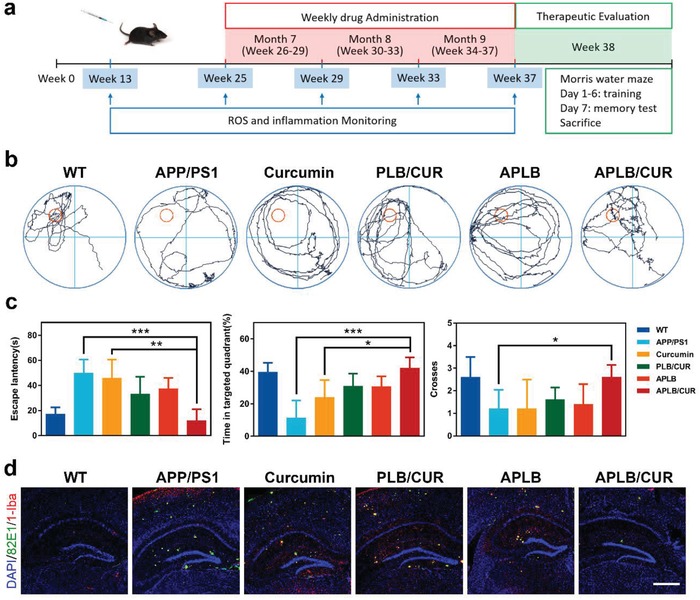
APLB/CUR improved memory and reduced Aβ burden in APP/PS1 mice. a) Time schedule of drug treatment, pathological monitoring, and therapeutic evaluation. b) Representative swimming paths of mice in Morris water maze. c) Escape latency, swimming time spent in the targeted quadrant and crosses over the platform site (error bars represent mean ± SD, *n* = 5). d) Immunostaining of mice hippocampus after 3‐month treatment (green: Aβ plaques, red: microglia, scale bar: 250 µm). *P* value in (c) was calculated by comparing with group APLB/CUR (*P< 0.05, **P< 0.01 or ***P< 0.001).

APP/PS1 transgenic mice were applied as the animal model to further substantiate our strategy. Mice were pregrouped and treated with various curcumin formulations as illustrated (**Figure** **4**a). The treatment began from an early stage in the disease progression at the age of 6 months (Week 26) when Aβ plaques are reported to just start forming in the brain in this model.^34^ Saline, curcumin, or micelles were administrated via tail vein weekly for 3 months from Week 26 to Week 37. Morris water maze test was performed at Week 38 to quantify the memory and cognitive improvement. Transgenic mice treated with APLB/CUR showed significantly improved memory behavior in finding the platform in the water maze, which was almost comparable to wild type mice, in terms of escape latency, swimming time spent in the targeted quadrant as well as crosses over the platform site (Figure 4b,c). Accordingly, the least Aβ and inflammation burdens (Figure 4d) and normalized neuron density^35^ (Figure S11, Supporting Information) were found in the hippocampus of APLB/CUR treated mice after 3 month treatment. These results demonstrated that the spatial cognition and memory improvement was related with positive brain microenvironment changes including Aβ burdens and neuroinflammation. Because of the scheduled multiple injections, we also investigated the potential toxicity of the polymeric micelles after treatment. As shown in the H&E staining, no obvious organ injury was found in all groups which indicated favorable biocompatibility of our system (Figure S12, Supporting Information). Due to the multiple failure of Aβ antibodies in clinical trials since the last decades, not only the targets of AD pathologies but also the timing of treatment has been reconsidered for AD drug development.^11^ It is suggested that intervention should be taken before the breaking point where cognitive impairment begins and too much damage has been caused.[[qv: 3a]] Our data showed that this early treatment with multiple targets might have a better chance to control microglia states before the irreversible damage occurs.

### 2.6 Monitoring of Brain Microenvironment Changes During Treatment and Underlying Mechanisms

It is hypothesized that newly proliferated microglia would develop a dysfunctional phenotype in the abnormal AD microenvironment which sustain chronic neuroinflammation and oxidative stress in AD.^36^ In response, we supposed that microenvironment changes during treatment could have a direct role in microglia modulation. To understand the process of how APLB/CUR changed AD microenvironment, immunostaining assay and cytokine measurements were performed at the age of 3 and 6 months before treatment and each month during treatment (Week 29, 33, and 37) to monitor oxidative stress and neuroinflammation as two major markers. In addition, dot blot was also performed to investigate the changes of soluble Aβ oligomers in brains.

Consistent with the clinical observation in AD patients,^37^ we found significant oxidative damage already emerged in hippocampus and cortex from 3‐month old and persisted to 6‐month old, which was indicated with the green fluorescence of 8‐hydroxyguanosine (8‐OHG) staining, a modified base in oxidized DNA. On the other hand, obvious activation of microglia and enhanced cytokine expression were also observed, even without severe burden of Aβ plaques by the age of 6 months (Figure S13, Supporting Information). Several researches suggest that aberrant microglia activation precedes the emergence of protein aggregation during AD progression.^10^, ^38^ The level of soluble oligomers in brain is reported to increase in the first phase in APP/PS1 mice, which is correlated with the gene regulation of immune response.^39^ As shown in the dot blot assay, we found increased Aβ oligomers in the brains of 3‐month and 6‐month‐old transgenic mice (Figure S14, Supporting Information). In fact, oligomer forms of Aβ are more toxic to neurons and microglia than the visible fibrils in the late stage.^40^ This might explain the hyperreactive state of microglia and oxidative stress we observed in the early phase and implies the dominant role of microglia in disrupting brain microenvironment and triggering AD development, which strengthens our hypothesis to modulate microglia in an early stage.

**Figure 5:**
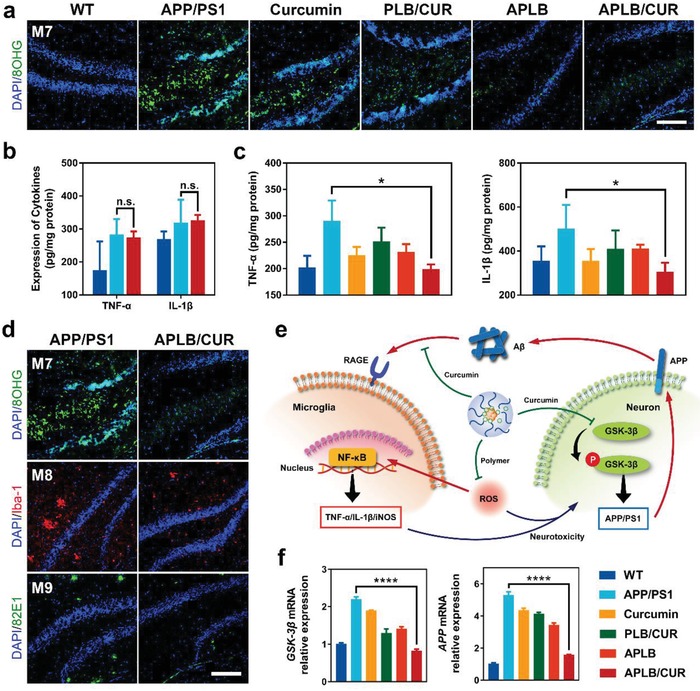
Mechanisms of APLB/CUR micelles modulation of microglia and AD microenvironment. a) Immunostaining of oxidative stress (green: 8‐OHG, scale bar: 100 µm) in the hippocampus of mice treated with different formulations for 1 month (M7). b) Brian cytokine expression after 1‐month treatment. c) Brian cytokine expression after 2‐month treatment. d) Immunostaining of mice hippocampus during different stages of treatment (blue: DAPI, green: 8‐OHG for oxidative damage or 82E1 for Aβ plaques, red: Iba‐1 for microglia, scale bar: 100 µm). e) Illustration of micelle modulation of two pathways for microglia activation. f) Relative expression of key proteins in the pathway of Aβ production in neurons. Error bars represent mean ± SD (*n* = 3). *P*‐value was calculated by comparing with group APLB/CUR (*P< 0.05 or ****P< 0.0001). Legends were the same for Figure 5b,c,f.

Microenvironment monitoring was followed up once the drug administration started. Surprisingly, significant relief of oxidative stress was first demonstrated after 1‐month (M7) treatment with APLB/CUR (**Figure** **5**a and Figure S15, Supporting Information). We assumed that micelles could immediately respond to the oxidative microenvironment and decrease the ROS level, due to its enhanced accumulation via RAGE targeting and quick ROS elimination by polymer degradation. The same phenomenon was also observed in mice treated with blank micelles, showing the dominant role of polymers in reducing oxidative stress. But even though oxidative damage was negligible at this time, massive gliosis could still be observed as demonstrated by the unchanged cytokine expression (Figure 5b) as well as the large number and shape of Iba‐1 microglia staining (Figure S16, Supporting Information). This was in accordance with the insignificant difference of oligomer levels between groups (Figure S14, Supporting Information). After 2‐month (M8) treatment, fewer activated microglia were found in APLB/CUR group (Figure S16), which was also substantiated by the decreased expression of tumor necrosis factor‐α (TNF‐α) and interleukin‐1β (IL‐1β) in the brain (Figure 5c,d). A slight decrease of cytokine expression was also observed in blank micelle treated mice compared with control group, indicating a weaker modulation of microglia without the participation of curcumin. At the end of 3‐month treatment (M9), mice injected with APLB/CUR showed least oxidative stress, microglia activation and cerebral burden of Aβ plaques, which was almost normalized as wild type mice (Figures S15 and S16, Supporting Information). Additionally, during treatment, a gradual decrease of Aβ oligomer level was found only in mice treated with APLB/CUR micelles (Figure S14, Supporting Information). Taken together, the microenvironment changes in AD brain after APLB/CUR treatment could be summarized into three steps as demonstrated in Figure 5d: 1) relieved oxidative stress first (M7); 2) followed by alleviated neuroinflammation (M8); and 3) eventually decreased Aβ burden (M9).

To understand the mechanisms of curcumin facilitated microglia modulation, we investigated the mRNA expression of several key proteins related with Aβ metabolism. As shown in Figure 5f and Figure S17 in the Supporting Information, we found significantly inhibited glycogen synthase kinase‐3β (GSK‐3β) expression and decreased APP, PS1 (γ‐secretase) mRNA expression in the brain of APLB/CUR treated mice. Elevated expression and activation of GSK‐3β has been reported in AD brains,^41^ which augments Aβ production by enhancing the transcription of amyloid cascade proteins including APP and PS1 (Figure 5e).^42^ Curcumin has been reported to downregulate GSK‐3β expression^43^ and directly bind Aβ oligomers to inhibit its toxicity which was also demonstrated in vitro (Fig 3c). Thus, we suggested curcumin could restrain Aβ‐induced microglia activation from both a biochemical level via Aβ inhibition and a transcriptional level by interrupting GSK‐3β pathway in neurons.

We suppose that the sequential changes of brain microenvironment during treatment reflect the responsiveness of microglia to APLB/CUR micelles. With the participation of multiple brain cells in AD progression, AD microenvironment is composed of various microglial activators including ROS and cytokines secreted from microglia themselves or damage associate molecular pattern and Aβ oligomers released from damaged neurons.^44^ These molecules further amplify microglia activation and form the vicious circle which eventually causes the irreversible neuron loss in the late stage. Therefore, multiple targets in AD microenvironment should be simultaneously controlled for effective treatment.[[qv: 1b]] With the strong ROS scavenging ability of the polymer, oxidative stress could be easily adjusted in the first place to protect neurons and attenuate microglia activation, but the excessive expression of Aβ from neurons might provide an alternative pathway to activate microglia, motivating them to be more resistant to antioxidant modulation. Our data showed that synergistic effects of carrier‐mediated ROS elimination and cargo mediated Aβ inhibition were required for microglia modulation, which might explain the difference between APLB and APLB/CUR on neuroinflammation inhibition. By normalizing AD microenvironment, Aβ burden could be reduced which in turn affected the metabolisms of damaged neurons and interdicted the vicious circle. These results further provide evidence that microglia could be modulated by targeting AD microenvironment with nanoparticles.

## 3 Conclusion

In summary, we have developed a curcumin‐loaded ROS‐responsive micelle system with the abilities of AD brain accumulation and subsequent microglia‐based microenvironment modulation. Through the synergistic effect of polymer and payloads, multiple targets in AD microenvironment could be controlled to reeducate the hyperactive microglia and protect damaged neurons, which show promising therapeutic effects in APP/PS1 model mice. Compared with the current nanoparticle‐based AD strategies, our system has several merits. First, by targeting to abnormal microglia apart from Aβ or tau aggregation, our treatment started from a rather early phase before the irreversible neuronal damage occurred, which might have a better potential for clinical translation. Second, with the RAGE targeting ability and the synergistic effect of polymer and cargo in the APLB/CUR micelles, our system could simultaneously eliminate extracellular ROS and soluble Aβ oligomers in AD microenvironment as well as intracellular ROS in both microglia and neurons. This multitarget strategy corresponds to the multicell interacting character of AD and could have more effective control of AD microenvironment compared with other single‐target systems.

The implications of this study on the future design of AD therapy are evident: the abnormality of microglia activity precedes other AD pathologies and early correction of microenvironment has a more profound impact on multiple cellular interactions, which is more effective than amyloid clearance in the rather late stage. Besides, this microglia‐centered drug delivery system could also be applied to other neuroinflammation associated diseases due to the crucial role of microglia in brain homeostasis.

## Conflict of Interest

The authors declare no conflict of interest.

## Supporting information

Supplementary

## References

[1] a) H. W. Querfurth , F. M. LaFerla , N. Engl. J. Med. 2010, 362, 329; 20107219 10.1056/NEJMra0909142

[2] a) C. Faustino , P. Rijo , C. P. Reis , Pharmacol. Res. 2017, 120, 68; 28351757 10.1016/j.phrs.2017.03.020

[3] a) G. Miller , Science 2012, 337, 790; 22903991 10.1126/science.337.6096.790

[4] a) T. M. Weitz , T. Town , Immunity 2016, 45, 717; 27760336 10.1016/j.immuni.2016.10.006

[5] D. Gosselin , D. Skola , N. G. Coufal , I. R. Holtman , J. C. M. Schlachetzki , E. Sajti , B. N. Jaeger , C. O Connor , C. Fitzpatrick , M. P. Pasillas , M. Pena , A. Adair , D. D. Gonda , M. L. Levy , R. M. Ransohoff , F. H. Gage , C. K. Glass , Science 2017, 356, eaal3222. 28546318 10.1126/science.aal3222 PMC5858585

[6] A. Niraula , J. F. Sheridan , J. P. Godbout , Neuropsychopharmacology 2017, 42, 318. 27604565 10.1038/npp.2016.185 PMC5143497

[7] D. Krstic , I. Knuesel , Nat. Rev. Neurol. 2013, 9, 25. 23183882 10.1038/nrneurol.2012.236

[8] a) Q. Song , M. Huang , L. Yao , X. Wang , X. Gu , J. Chen , J. Chen , J. Huang , Q. Hu , T. Kang , Z. Rong , H. Qi , G. Zheng , H. Chen , X. Gao , ACS Nano 2014, 8, 2345; 24527692 10.1021/nn4058215

[9] F. Mangialasche , A. Solomon , B. Winblad , P. Mecocci , M. Kivipelto , Lancet Neurol. 2010, 9, 702. 20610346 10.1016/S1474-4422(10)70119-8

[10] S. Hong , V. F. Beja‐Glasser , B. M. Nfonoyim , A. Frouin , S. Li , S. Ramakrishnan , K. M. Merry , Q. Shi , A. Rosenthal , B. A. Barres , C. A. Lemere , D. J. Selkoe , B. Stevens , Science 2016, 352, 712. 27033548 10.1126/science.aad8373 PMC5094372

[11] F. L. Heppner , R. M. Ransohoff , B. Becher , Nat. Rev. Neurosci. 2015, 16, 358. 25991443 10.1038/nrn3880

[12] D. Gackowski , R. Rozalski , A. Siomek , T. Dziaman , K. Nicpon , M. Klimarczyk , A. Araszkiewicz , R. Olinski , J. Neurol. Sci. 2008, 266, 57. 17888453 10.1016/j.jns.2007.08.041

[13] M. Rosini , E. Simoni , A. Milelli , A. Minarini , C. Melchiorre , J. Med. Chem. 2014, 57, 2821. 24131448 10.1021/jm400970m

[14] D. J. Bonda , X. Wang , G. Perry , A. Nunomura , M. Tabaton , X. Zhu , M. A. Smith , Neuropharmacology 2010, 59, 290. 20394761 10.1016/j.neuropharm.2010.04.005

[15] T. Taetzsch , S. Levesque , C. McGraw , S. Brookins , R. Luqa , M. G. Bonini , R. P. Mason , U. Oh , M. L. Block , Glia 2015, 63, 423. 25331559 10.1002/glia.22762 PMC4322433

[16] a) H. J. Kwon , M. Cha , D. Kim , D. K. Kim , M. Soh , K. Shin , T. Hyeon , I. Mook‐Jung , ACS Nano 2016, 10, 2860; 26844592 10.1021/acsnano.5b08045

[17] W. A. Banks , Adv. Drug Delivery Rev. 2012, 64, 629. 10.1016/j.addr.2011.12.005 PMC3389492 22202501

[18] a) L. Lue , D. G. Walker , L. Brachova , T. G. Beach , J. Rogers , A. M. Schmidt , D. M. Stern , S. D. Yan , Exp. Neurol. 2001, 171, 29; 11520119 10.1006/exnr.2001.7732

[19] R. Deane , S. Du Yan , R. K. Submamaryan , B. LaRue , S. Jovanovic , E. Hogg , D. Welch , L. Manness , C. Lin , J. Yu , H. Zhu , J. Ghiso , B. Frangione , A. Stern , A. M. Schmidt , D. L. Armstrong , B. Arnold , B. Liliensiek , P. Nawroth , F. Hofman , M. Kindy , D. Stern , B. Zlokovic , Nat. Med. 2003, 9, 907. 12808450 10.1038/nm890

[20] B. P. Cary , A. F. Brooks , M. V. Fawaz , L. R. Drake , T. J. Desmond , P. Sherman , C. A. Quesada , P. J. H. Scott , ACS Chem. Neurosci. 2016, 7, 391. 26771209 10.1021/acschemneuro.5b00319 PMC5682588

[21] M. Mehanny , R. M. Hathout , A. S. Geneidi , S. Mansour , J. Controlled Release 2016, 225, 1. 10.1016/j.jconrel.2016.01.018 26778694

[22] K. E. Broaders , S. Grandhe , J. M. J. Fréchet , J. Am. Chem. Soc. 2011, 133, 756. 21171594 10.1021/ja110468v

[23] E. Gospodarska , A. Kupniewska‐Kozak , G. Goch , M. Dadlez , Biochim. Biophys. Acta, Proteins Proteomics 2011, 1814, 592. 10.1016/j.bbapap.2011.02.011 21354340

[24] C. de Gracia Lux , S. Joshi‐Barr , T. Nguyen , E. Mahmoud , E. Schopf , N. Fomina , A. Almutairi , J. Am. Chem. Soc. 2012, 134, 15758. 22946840 10.1021/ja303372u PMC3478073

[25] Y. Zhang , Q. Guo , S. An , Y. Lu , J. Li , X. He , L. Liu , Y. Zhang , T. Sun , C. Jiang , ACS Appl. Mater. Interfaces 2017, 9, 12227. 28350451 10.1021/acsami.6b16815

[26] J. Kwon , J. Kim , S. Park , G. Khang , P. M. Kang , D. Lee , Biomacromolecules 2013, 14, 1618. 23590189 10.1021/bm400256h

[27] H. Xie , S. Hou , J. Jiang , M. Sekutowicz , J. Kelly , B. J. Bacskai , Proc. Natl. Acad. Sci. USA 2013, 110, 7904. 23610434 10.1073/pnas.1217938110 PMC3651444

[28] A. I. Rojo , G. McBean , M. Cindric , J. Egea , M. G. López , P. Rada , N. Zarkovic , A. Cuadrado , Antioxid. Redox Signaling 2014, 21, 1766. 10.1089/ars.2013.5745 PMC4186766 24597893

[29] a) B. Kuhla , C. Loske , S. Garcia De Arriba , R. Schinzel , J. Huber , G. M Nch , J. Neural Transm. 2004, 111, 427; 14991463 10.1007/s00702-003-0038-2

[30] S. Raha , H. J. Lee , S. Yumnam , G. E. Hong , G. S. V. Venkatarame , Y. L. Ha , J. O. Kim , Y. S. Kim , J. D. Heo , S. J. Lee , E. H. Kim , G. S. Kim , Life Sci. 2016, 161, 37. 27477351 10.1016/j.lfs.2016.07.017

[31] a) M. Wei , L. Chen , J. Liu , J. Zhao , W. Liu , F. Feng , Neurosci. Lett. 2016, 617, 143; 26876445 10.1016/j.neulet.2016.02.019

[32] Y. Zhao , B. Zhao , Oxid. Med. Cell. Longevity 2013, 2013, 1. 10.1155/2013/316523 PMC3745981 23983897

[33] A. Belkacemi , S. Doggui , L. Dao , C. Ramassamy , Expert Rev. Mol. Med. 2011, 13, e34. 22051121 10.1017/S1462399411002055

[34] A. A. Babcock , L. Ilkjær , B. H. Clausen , B. Villadsen , L. Dissing‐Olesen , A. T. M. Bendixen , L. Lyck , K. L. Lambertsen , B. Finsen , Brain, Behav., Immun. 2015, 48, 86. 25774009 10.1016/j.bbi.2015.03.006

[35] G. S. Chai , Y. Y. Wang , A. Yasheng , P. Zhao , Neural Regener. Res. 2016, 11, 1617. 10.4103/1673-5374.193241 PMC5116841 27904493

[36] M. T. Heneka , D. T. Golenbock , E. Latz , Nat. Immunol. 2015, 16, 229. 25689443 10.1038/ni.3102

[37] A. Nunomura , G. Perry , G. Aliev , K. Hirai , A. Takeda , E. K. Balraj , P. K. Jones , H. Ghanbari , T. Wataya , S. Shimohama , S. Chiba , C. S. Atwood , R. B. Petersen , M. A. Smith , J. Neuropathol. Exp. Neurol. 2001, 60, 759. 11487050 10.1093/jnen/60.8.759

[38] A. L. Wright , R. Zinn , B. Hohensinn , L. M. Konen , S. B. Beynon , R. P. Tan , I. A. Clark , A. Abdipranoto , B. Vissel , PLoS One 2013, 8, e59586. 23560052 10.1371/journal.pone.0059586 PMC3613362

[39] I. Lopez‐Gonzalez , A. Schluter , E. Aso , P. Garcia‐Esparcia , B. Ansoleaga , F. LLorens , M. Carmona , J. Moreno , A. Fuso , M. Portero‐Otin , R. Pamplona , A. Pujol , I. Ferrer , J. Neuropathol. Exp. Neurol. 2015, 74, 319. 25756590 10.1097/NEN.0000000000000176

[40] I. Benilova , E. Karran , B. De Strooper , Nat. Neurosci. 2012, 15, 349. 22286176 10.1038/nn.3028

[41] Y. Feng , Y. Xia , G. Yu , X. Shu , H. Ge , K. Zeng , J. Wang , X. Wang , J. Neurochem. 2013, 126, 234. 23646926 10.1111/jnc.12285

[42] a) P. Picone , D. Nuzzo , L. Caruana , E. Messina , A. Barera , S. Vasto , M. Di Carlo , Biochim. Biophys. Acta, Mol. Cell Res. 2015, 1853, 1046; 10.1016/j.bbamcr.2015.01.017 25667085

[43] X. Zhang , W. Yin , X. Shi , Y. Li , Eur. J. Pharm. Sci. 2011, 42, 540. 21352912 10.1016/j.ejps.2011.02.009

[44] M. L. Block , L. Zecca , J. Hong , Nat. Rev. Neurosci. 2007, 8, 57. 17180163 10.1038/nrn2038

