# Supplementary material for: Microenvironment Remodeling Micelles for Alzheimer's Disease Therapy by Early Modulation of Activated Microglia
Source: Adv Sci (Weinh). 2018 Dec 12;6(4):1801586. doi: 10.1002/advs.201801586 (PMC6382295; doi:10.1002/advs.201801586)
Supplement: Supplementary file 1 — Supplementary [file ADVS-6-1801586-s001.pdf]

Copyright WILEY-VCH Verlag GmbH & Co. KGaA, 69469 Weinheim, Germany, 2018.

ADVANCED SCIENCE
Open Access

Supporting Information

for *Adv. Sci.,* DOI: 10.1002/advs.201801586

Microenvironment Remodeling Micelles for Alzheimer's Disease Therapy by Early Modulation of Activated Microglia

*Yifei Lu, Zhongyuan Guo, Yujie Zhang, Chao Li, Yu Zhang, Qin Guo, Qinjun Chen, Xinli Chen, Xi He, Lisha Liu, Chunhui Ruan, Tao Sun, Bin Ji, Weigen Lu, and Chen Jiang**

Copyright WILEY-VCH Verlag GmbH & Co. KGaA, 69469 Weinheim, Germany, 2016.

Supporting Information

Microenvironment remodeling micelles for Alzheimer's disease therapy by early modulation of activated microglia

*Yifei Lu, Zhongyuan Guo, Yujie Zhang, Chao Li, Yu Zhang, Qin Guo, Qinjun Chen, Xinli Chen, Xi He, Lisha Liu, Chunhui Ruan, Tao Sun, Bin Ji, Weigen Lu and Chen Jiang**

Contents:

Figure S1. Synthesis route of Ab-PEG-pLysB (APLB) polymer

Figure S2. ^{1}H NMR and GPC characterization of polymers

Figure S3. IR characterization of Ab-PEG-pLysB

Figure S4. HPLC result of degraded PEG-pLysB polymer

Figure S5. TEM images of PLB/CUR micelles and APLB micelles

Figure S6. CMC determination of APLB/CUR

Figure S7. Cellular uptake of APLB/CUR with different Ab peptide modification

Figure S8. Ab peptide modification facilitated micelle transport across BBB in vitro

Figure S9. RAGE expression in APP/PS1 transgenic mice.

Figure S10. Biodistribution study of APLB/BODIPY micelles in WT and APP/PS1 mice.

Figure S11. Neuron density in the hippocampus after three-month treatment

Figure S12. H&E staining results of major organs after 3-month treatment

Figure S13. Brain microenvironment in early stage of AD progression

Figure S14. Brain level of soluble Aβ oligomers

Figure S15. Alleviation of oxidative stress in AD brain

Figure S16. Alleviation of neuroinflammation and Aβ burdens in AD brain

Figure S17. Relative expression of key proteins in the pathway of Aβ production

Table S1. Summary of micelle properties

Experimental section

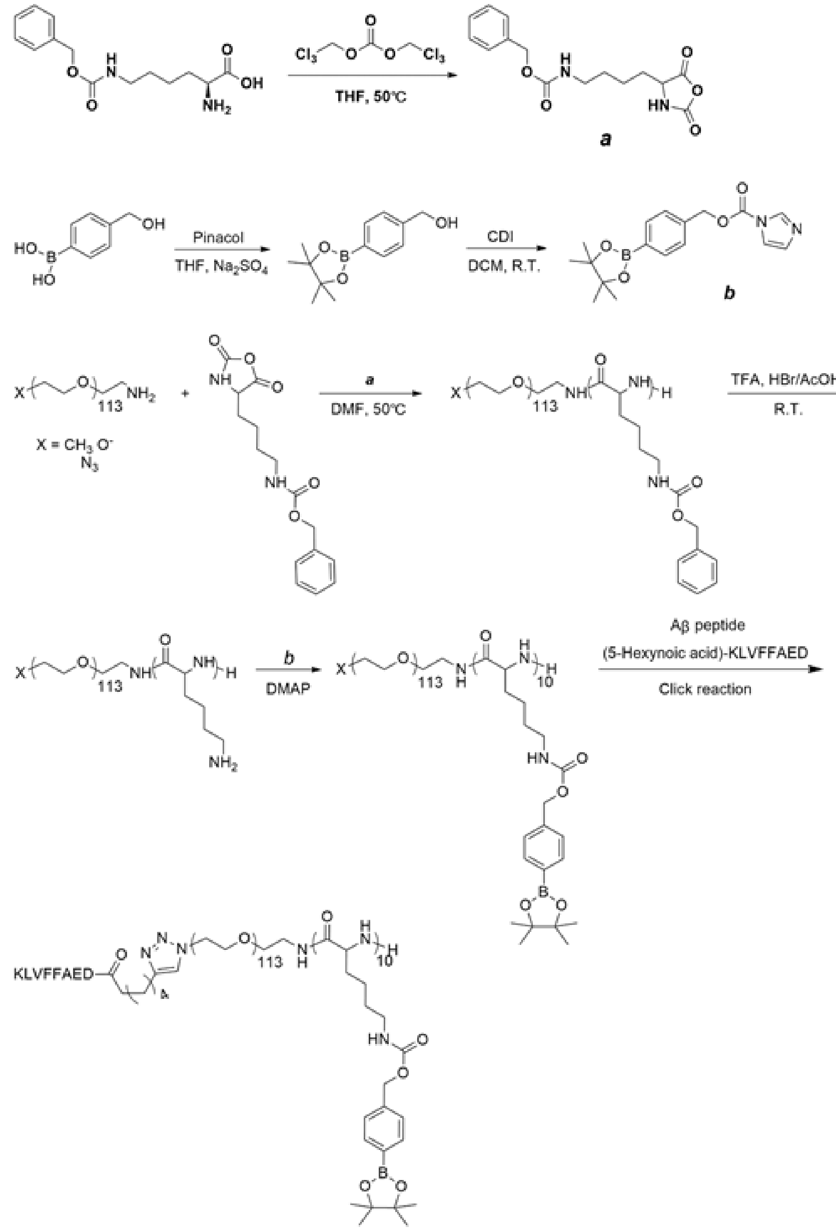

Figure S1. Synthesis route of Ab-PEG-pLysB (APLB) polymer.

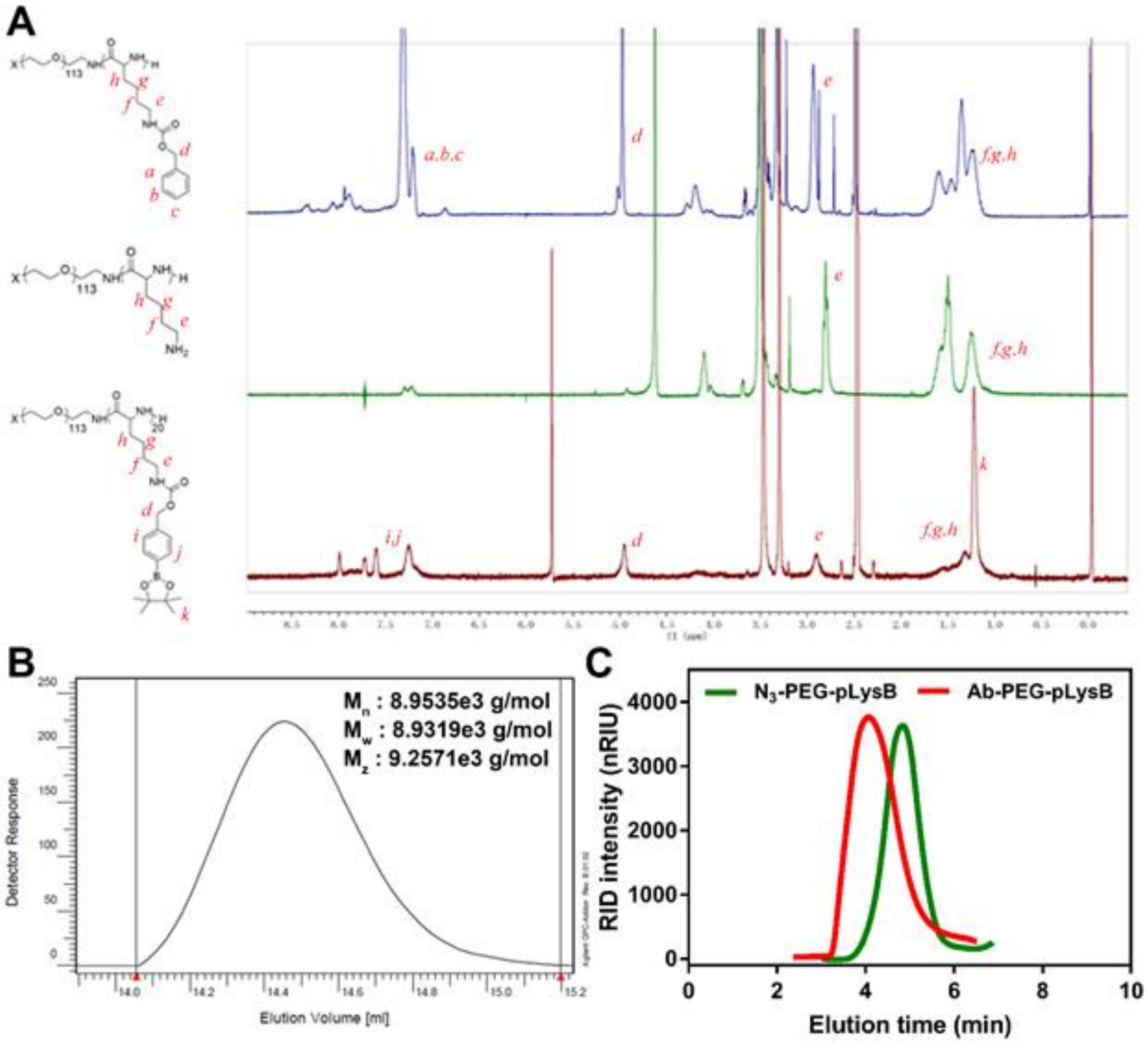

Figure S2. (A)^{1}H NMR spectrum of PEG-pLys(*Z*) (top), PEG-pLys (middle), PEG-pLysB (bottom); (B) GPC result and calculated molecule weight of PEG-pLysB; (C) GPC elution time of N_3-PEG-pLysB and Ab-EPG-pLysB.

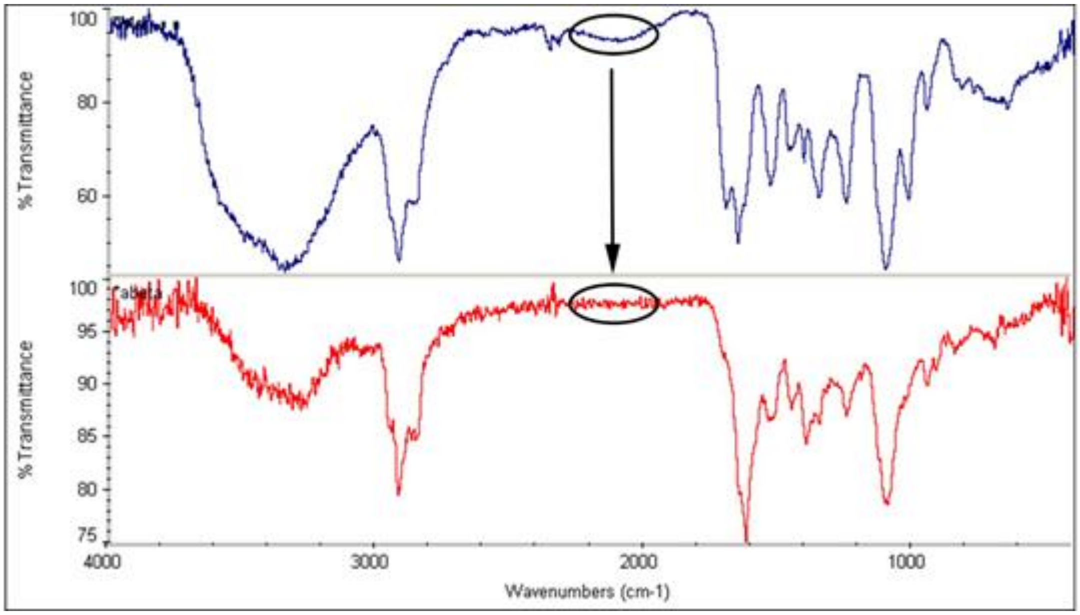

Figure S3. IR spectrum of N_3-PEG-pLysB (top) and Ab-PEG-pLysB (bottom), the arrow indicates the disappearance of azide group at 2100 cm^{-1} after Ab peptide conjugation.

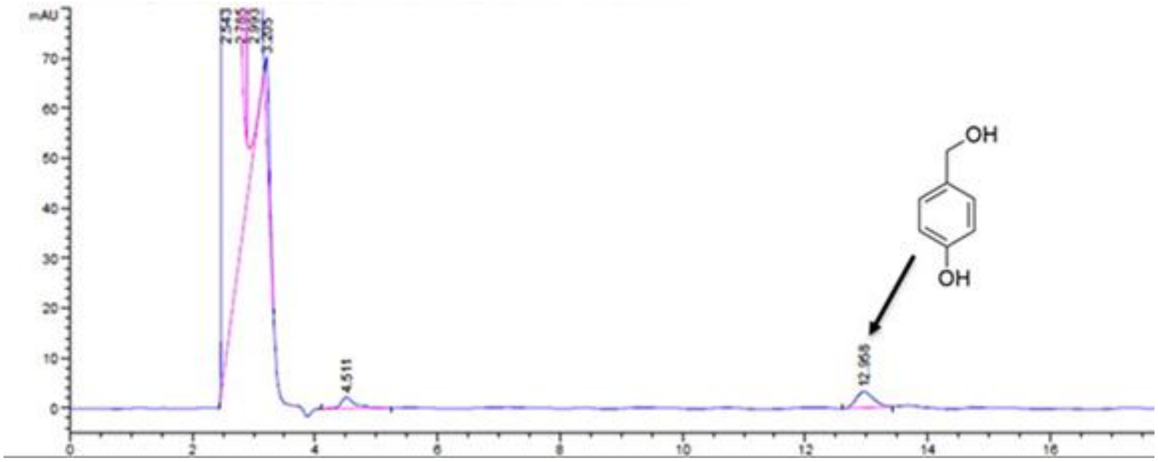

Figure S4. HPLC result of degraded PEG-pLysB polymer after incubation with 100 μM H_2O_2. The peak at 12.968 min indicates the degradation product 4-Hydroxybenzylalcohol.

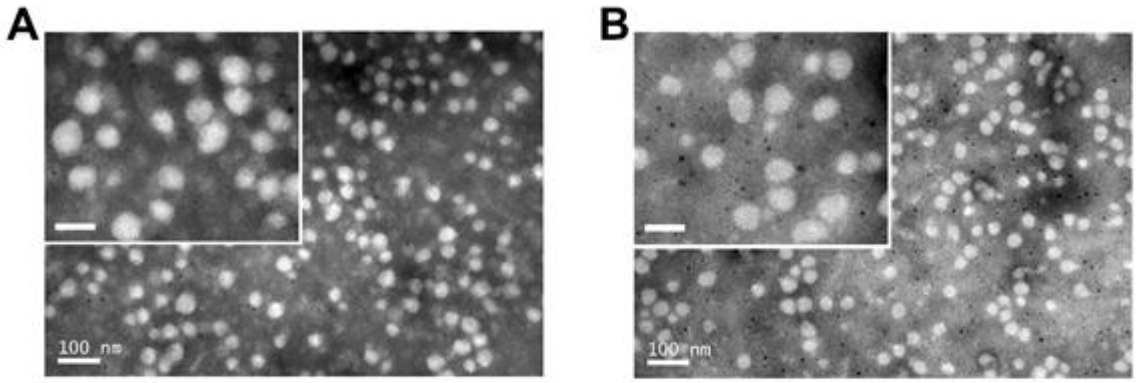

Figure S5. TEM images of PLB/CUR micelles(a) and APLB micelles(b) (scale bar: 100 nm; inset scale bar: 50 nm).

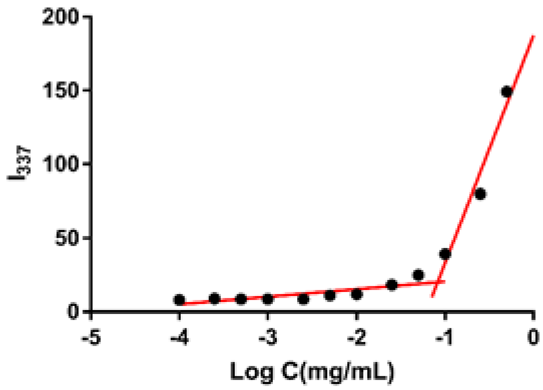

Figure S6. CMC determination of APLB/CUR. The CMC is defined as the point of intersection of two exponential lines shown in red lines.

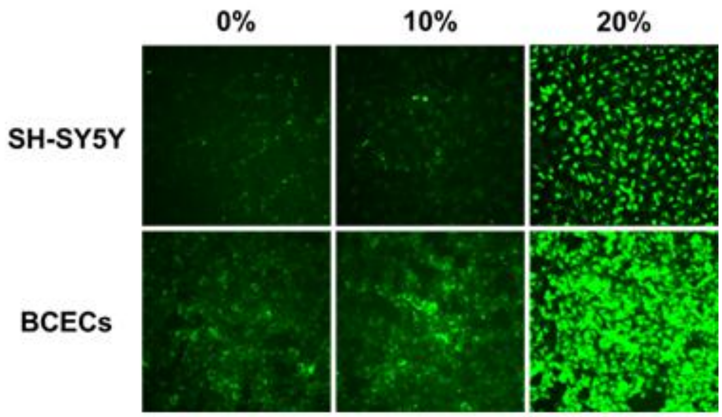

Figure S7. Cellular uptake of APLB/CUR with different Ab peptide modification in SH-SY5Y and BCECs. 20% modification of Ab peptide was applied in all the other experiments.

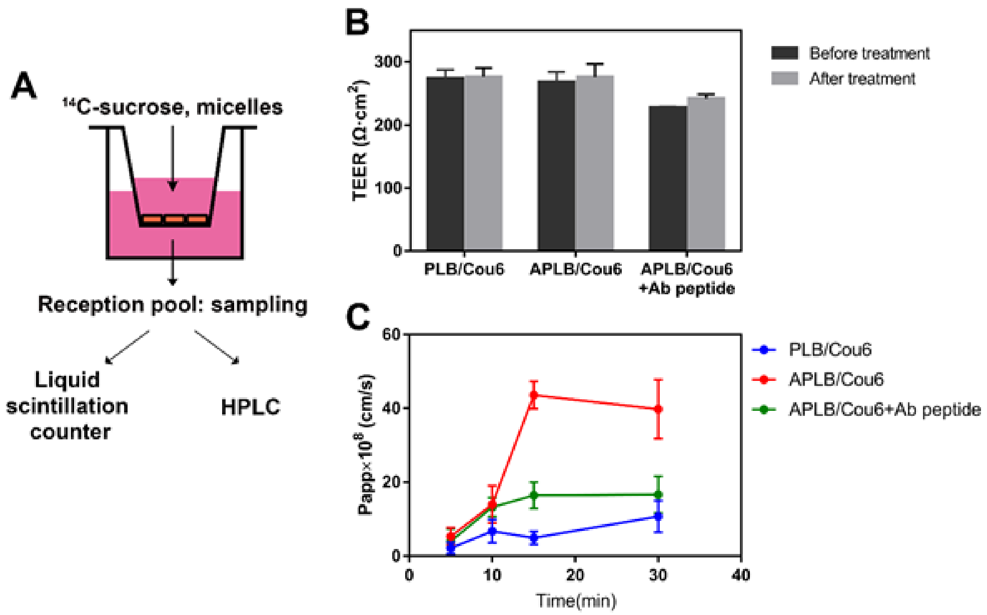

Figure S8. Ab peptide modification facilitated micelle transport across BBB in vitro. (a) Illustration of the BCECs monolayer transwell system. Permeability to ^{14}C-sucrose in three groups were all smaller than 8.0×10^{-5} cm/s as previously reported by others (*Brain Res*. **2006**, 1109, 1-13). (b) TEER measurement before and after the experiment. (c) The permeability of PLB/Cou6 and APLB/Cou6 micelles with or without free peptide preincubation. Results are reported as mean±SEM (n=3).

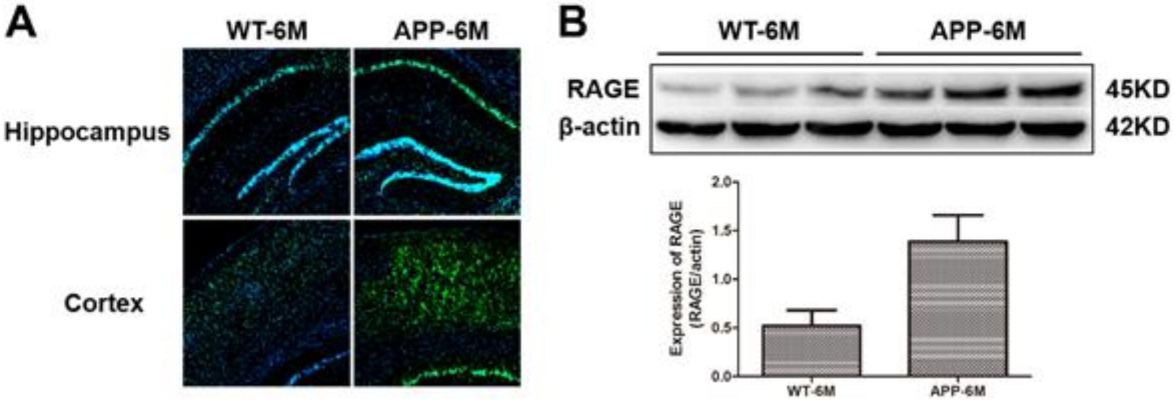

Figure S9. RAGE expression in APP/PS1 transgenic mice. (A) Representative immunofluorescence images of mice hippocampus and cortex. Brains were immunostained for RAGE (in green) and DAPI nuclear staining. (B) Western blot analysis of RAGE protein levels in the brains of 6 months old wild type (WT-6M) and APP/PS1 (APP-6M) mice (top) and quantitation result (bottom).

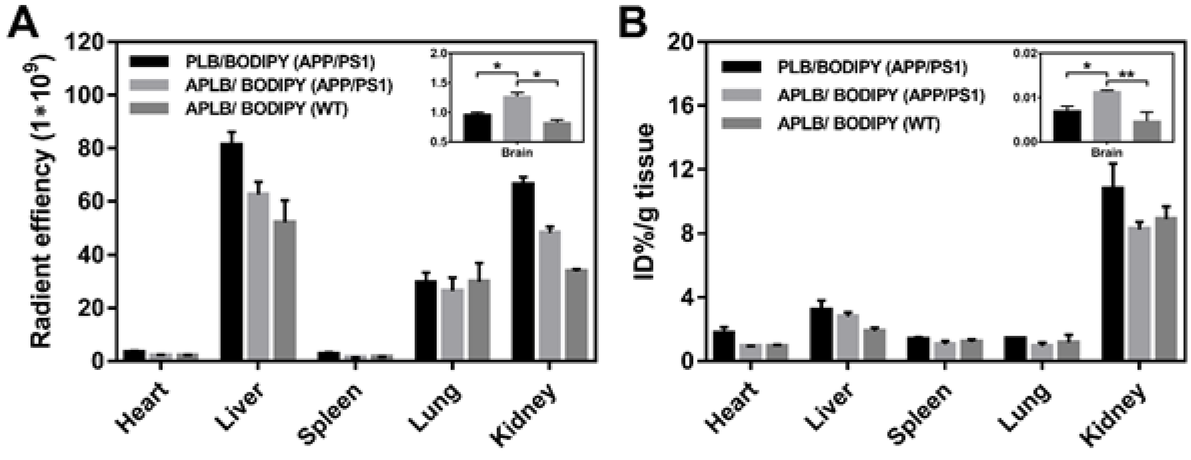

Figure S10. Biodistribution study of APLB/BODIPY micelles in WT and APP/PS1 mice. (A) Radiant efficiency of BODIPY fluorescence intensity in major organs and brain (insert) 4 h post-injection. (B) ID% analysis of BODIPY accumulation in major organs and brain (insert) 4 h post-injection. Results are reported as mean±SEM (n=3, *$P<0.05$ or **$P<0.01$).

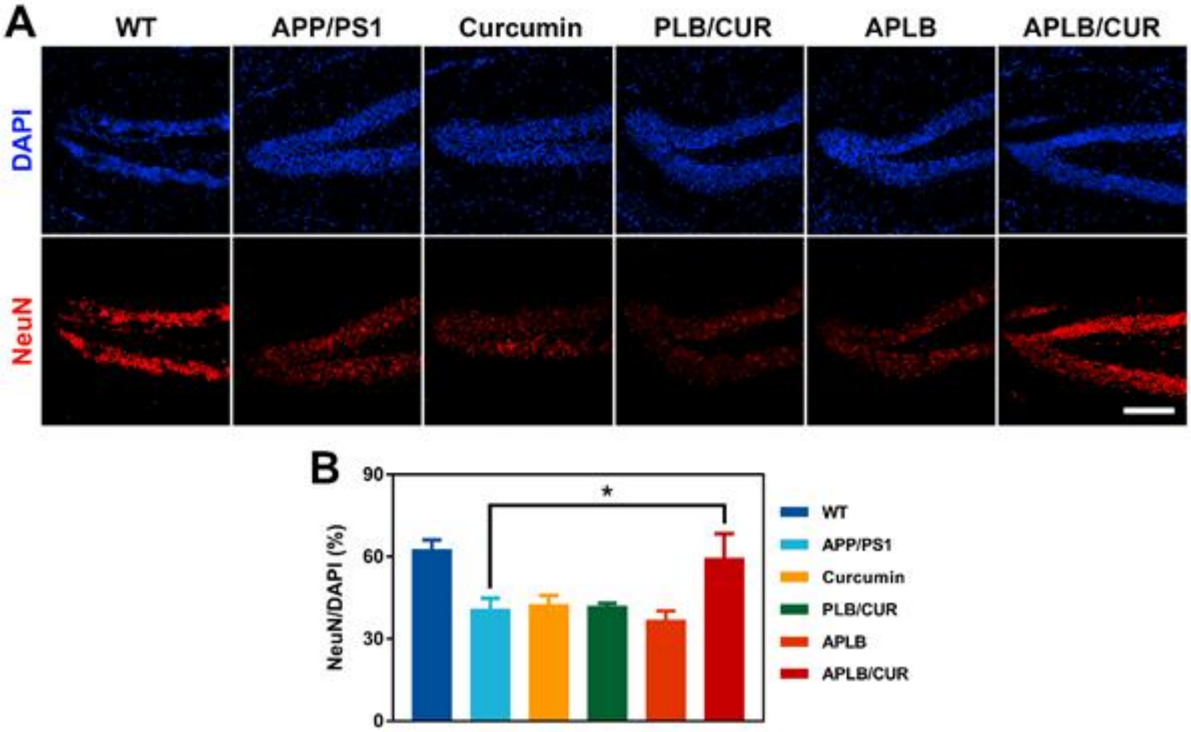

Figure S11. Neuron density in the hippocampus after three-month treatment. (a) Immunostaining of hippocampal DG neurons (NeuN-positive cells, red); scale bars: 100 μm. (b) Quantification for the ratio of NeuN and DAPI positive nuclei in a defined area; n=3 images from 2 independent slices; results are reported as mean±SD. (**P*<0.05)

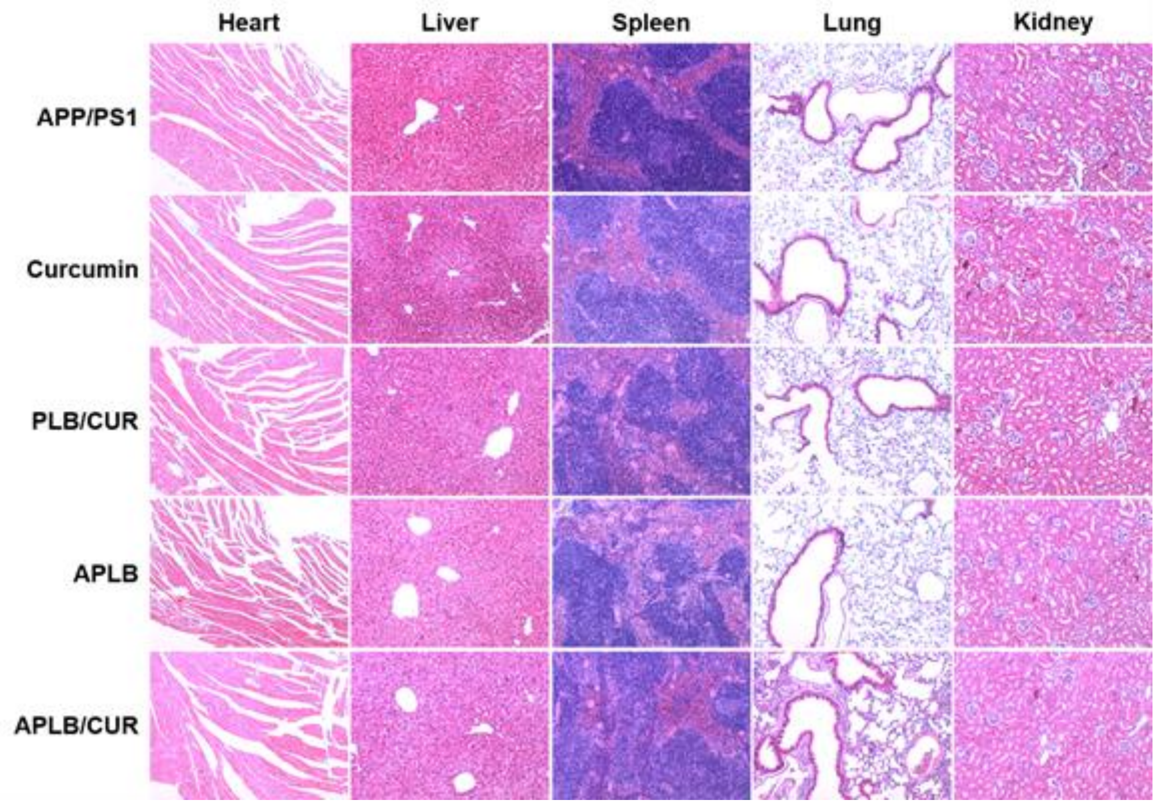

Figure S12. H&E staining results of major organs after 3-month treatment of different formulations.

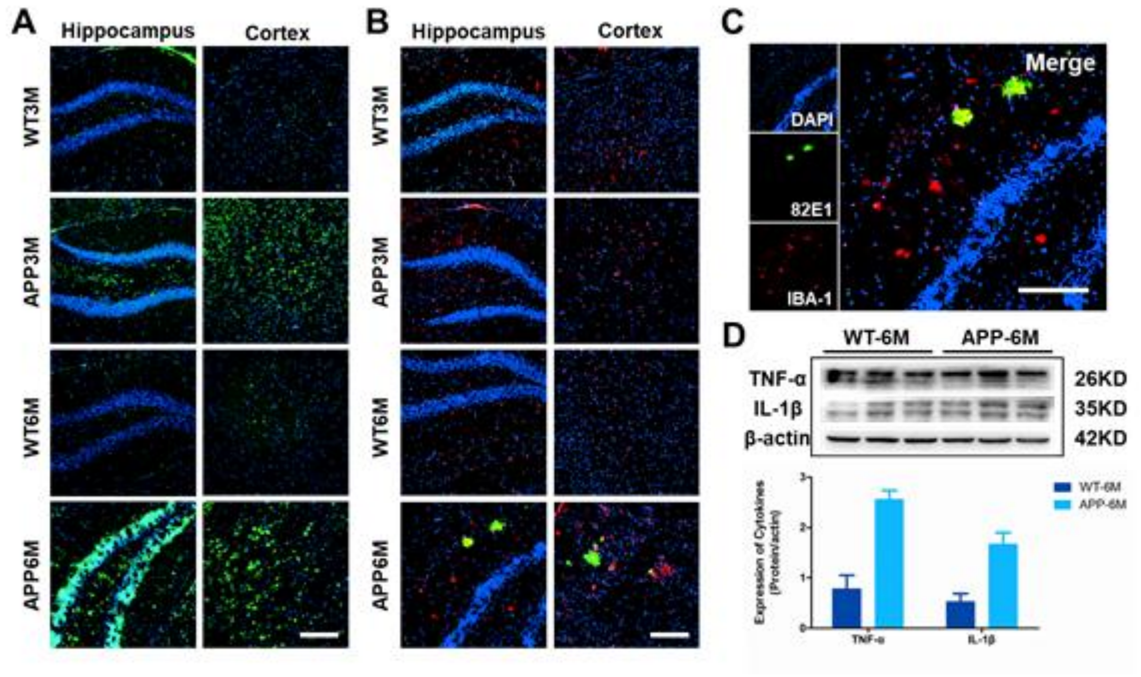

Figure S13. Brain microenvironment in early stage of AD progression. Brain sections of 3-month-old (3M) and 6-month-old (6M) wild type (WT) and APP/PS1 (APP) mice were applied for the immunoassays. (A) Immunostaining of oxidative stress (8-OHG, green). (B) Immunostaining of neuroinflammation (Iba-1 for microglia, red; 82E1 for Aβ plaques, green). (C) Magnification of the hippocampus of APP-6M mouse in **B**. (D) Western blot analysis of TNF-α and IL-1β protein levels in the brains of WT-6M and APP-6M mice (top) and quantitation result (bottom). Scale bars in B,C: 100 μm.

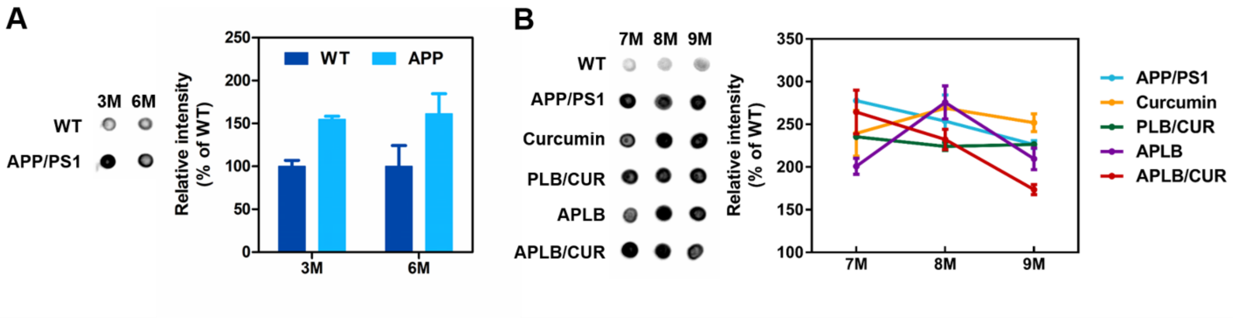

Figure S14. Brain level of soluble Aβ oligomers in early stage of AD progression and during treatment. (A) Dot blot analysis of Aβ oligomer levels in the brains of WT-3M/APP-3M and WT-6M/APP-6M mice (left) and quantitation result (right). (B) Dot blot analysis of Aβ oligomer levels in the brains of WT and APP mice treated with different formulations (left) and quantitation result (right). Results are reported as mean±SEM (n=2).

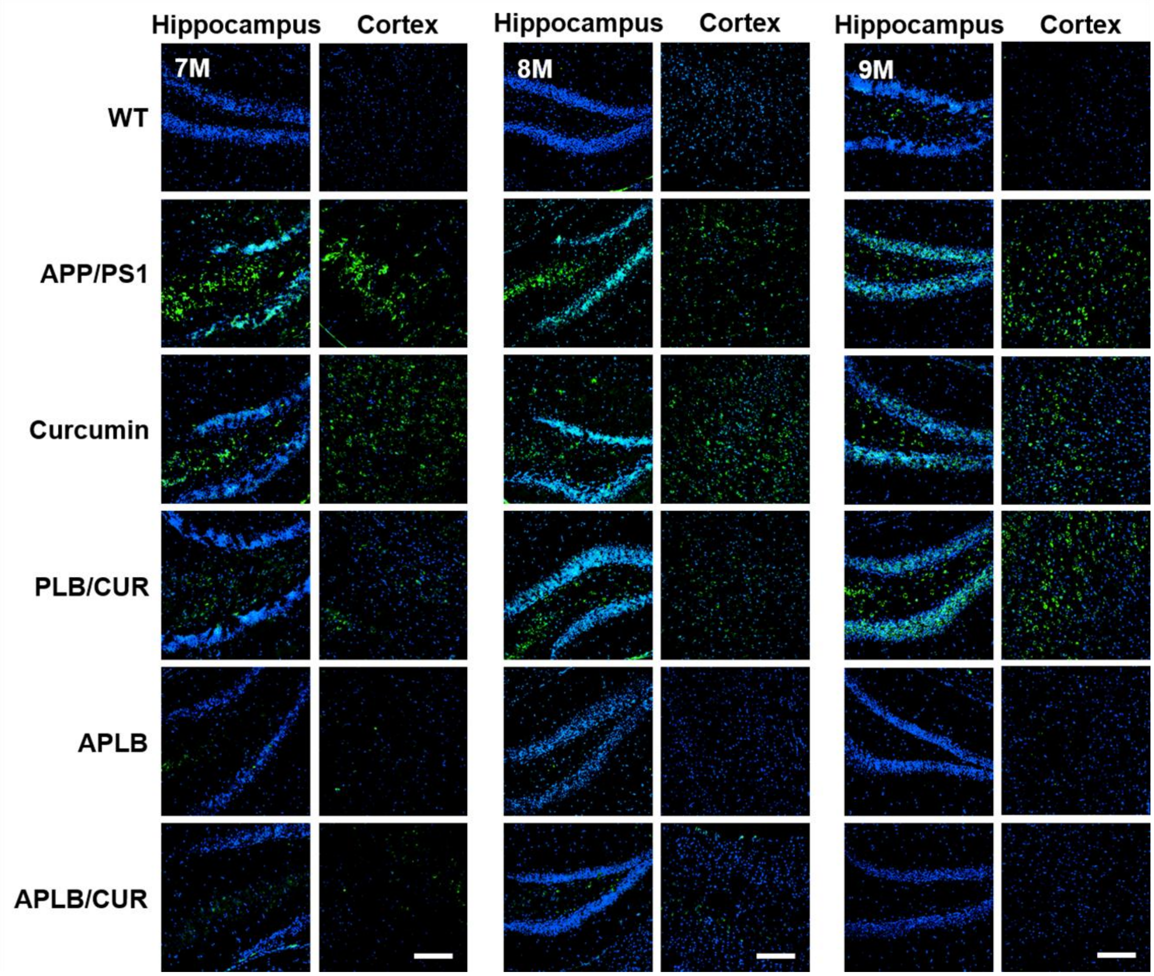

Figure S15. Alleviation of oxidative stress in AD brain during treatment. Immunostaining of oxidative stress (8-OHG, green) in the hippocampus and cortex of mice treated with different formulations for one month(7M), two months(8M) and three months(9M). Scale bars: 100 μm.

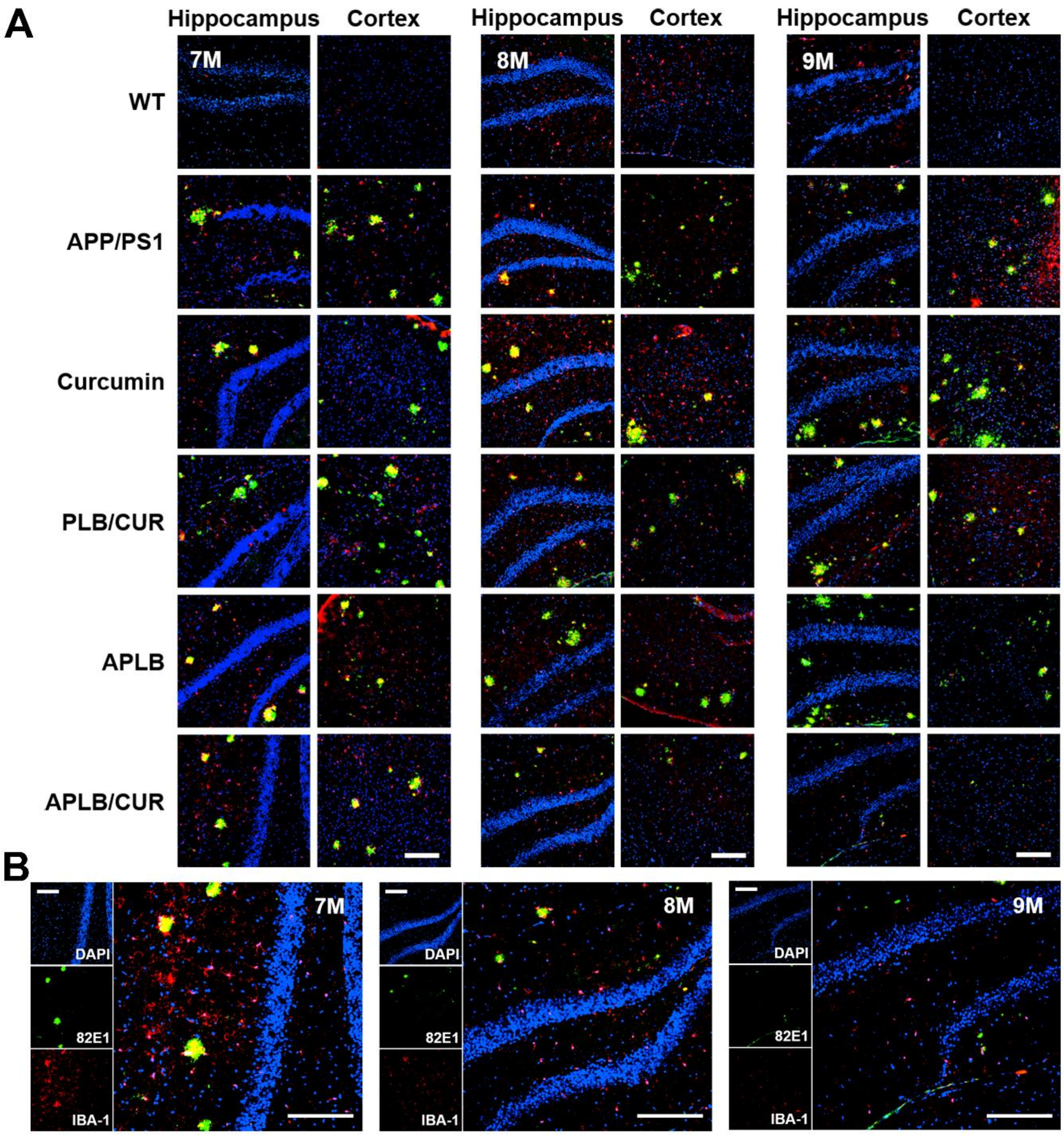

Figure S16. Alleviation of neuroinflammation and Aβ burdens in AD brain during treatment. (A) Immunostaining of neuroinflammation (Iba-1 for microglia, red; 82E1 for Aβ plaques, green) in the hippocampus and cortex of mice treated with different formulations for one month(7M), two months(8M) and three months(9M). (B) Magnification of the hippocampus of APLB/CUR treated mice in **A**. Scale bars: 100 μm.

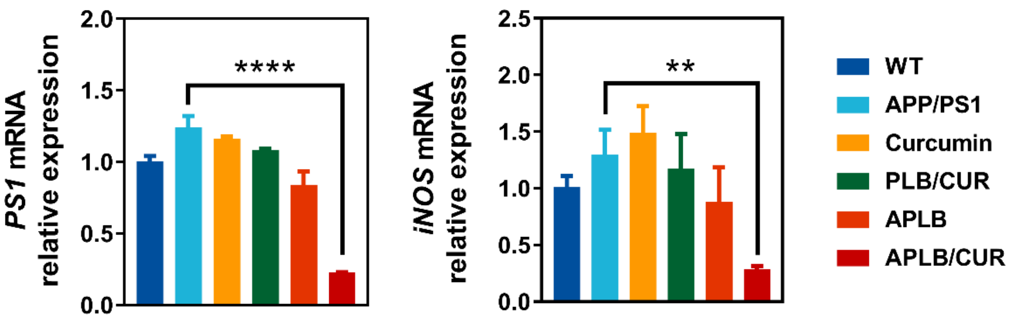

Figure S17. Relative expression of key proteins in the pathway of Aβ production in neurons. Error bars represent mean ± SD (n=3). *P* value was calculated by comparing with group APLB/CUR (**P*<0.05 or *****P*<0.0001).

Table S1. Summary of micelle properties.

Polymeric micelles	Size [*d*, nm]	PDI	Zeta potential [mV]	EE%[a)]	DL%[b)]
PLB/CUR	65.5±0.2	0.07±0.01	-2.22±0.34	95.3±5.9%	8.7±0.5%
APLB	59.5±0.3	0.08±0.00	0.03±0.08	-	-
APLB/CUR	63.0±0.5	0.09±0.02	2.54±0.19	91.4±4.8%	8.3±0.4%

[a)]Encapsulation efficiency (EE%): ratio of encapsulated drug to total drug used for micelle preparation.

[b)]Drug loading (DL%): ratio of encapsulated drug to the total mass of micelles.

Experimental section

Materials. α-Methoxy-ω-amino-poly(ethylene glycol) (mPEG-NH_2, Mw 5000) was purchased from Seebio Biotech (Shanghai, China). α-Azide-ω-amino-poly(ethylene glycol) (N_3-PEG-NH_2, Mw 5000) was purchased from Jenkem Technology (Beijing, China). Ab peptide (sequence: 5-Hexynoic acid-KLVFFAED) was synthesized by ChinaPeptides Co. Ltd (Nanjing, China). Curcumin was purchased from J&K Chemical (Beijing, China). All the other chemicals were purchased from Energy Chemical (Shanghai, China).

Coumarin, phenylarsine oxide (PhAsO), colchicine, filipin, 3-(4,5-dimethyl-2-thiazolyl)-2,5-diphenyl-2H tetrazolium bromide (MTT) and 4',6-diamidino-2-phenylindole (DAPI) were purchased from Sigma-Aldrich (St. Louis, USA). Hoechst 33342 trihydrochloride, LysoTracker Deep Red and BODIPY (650/665) were purchased from Molecular Probes (Waltham, USA). Amplite™ Fluorometric Hydrogen Peroxide Assay Kit was purchased from AAT Bioquest (California, USA). Annexin V-FITC/PI apoptosis detection kit were purchased from KeyGEN BioTECH (Nanjing, China). Fluorescence probe dihydroethidium (DHE) and Bradford protein quantification kit was purchased from Beyotime (Shanghai, China). All the antibodies and ELISA kits were purchased from Abcam (Shanghai, China). All the other reagents were purchased from Sinopharm Chemical Reagent (Shanghai, China).

Synthesis and characterization of Ab-PEG-pLysB (APLB) polymer. N^6-Carbobenzyloxy-L-lysine N-carboxyanhydride (Lys(*Z*)-NCA, Compound ***a***) *[55]* and imidazolyl carbamate of pinacol boronic ester (Compound ***b***) *[30]*was synthesized as reported previously. To obtain the polymer via ROP reaction, CH_3O-PEG-NH_2 or N_3-PEG-NH_2 (1g, 0.2 mmol) and Lys(*Z*)-NCA (2.4g, 8 mmol) was dissolved in anhydrous DMF and stirred at 50℃ for 2 days under argon. The mixture was dialyzed against deionized water and further lyophilized to yield PEG-pLys(*Z*) as a white solid. For deprotection of lysine, PEG-pLys(*Z*) was dissolved in 10 mL TFA and 0.5 mL HBr/HOAc, stirred at room temperature for 5 hours, followed by dialysis and lyophilization. The obtained solid (500 mg, 0.067 mmol) was then dissolved in DMF with Compound ***b*** (656mg, 2 mmol), 4-dimethylaminopyridine (DMAP, 243 mg, 2 mmol) and triethylamine (202mg, 2 mmol). The mixture was allowed stirring at room temperature for 2 days and then dialyzed against DMF for 12 hours and deionized water for another 24 hours before lyophilization to obtain PEG-pLysB.

Ab peptide was conjugated with N_3-PEG-pLysB via copper(Ⅰ) catalyzed Click reaction. In brief, N_3-PEG-pLysB (100 mg, 0.02 mmol) and alkyne-terminated peptide (0.04 mmol) was

dissolved in DMF, then excess sodium L-ascorbate (30 equiv), DIPEA (3 equiv) and CuI (3 equiv) was added to the solution under argon protection. The reaction was carried out overnight at room temperature and dialyzed against PBS 7.4 containing 10 mM EDTA for 12 hours and deionized water for another 24 hours before lyophilization.

All synthesized chemicals were characterized by ^{1}H NMR spectra using an NMR spectrometer (400 MHz, Bruker, Coventry, U.K.) or IR spectra (Thermo Scientific, Germany). Molecular weight of the polymer was measured by gel permeation chromatography (GPC, Polymer Laboratories Inc.). The degradation profile of the polymer was determined by measuring the degradation product 4-hydroxybenzylalcohol with HPLC. HPLC method was set as: Agilent C18 column, 250mm, 1.0 mL/min, 10% methanol + 90% 0.1% trifluoroacetic acid, injection volume 10 μL, VWD wave length: 220 nm. Briefly, polymer was dissolved in methanol and further diluted with 10 mM PBS 7.4 or 10 mM PBS 7.4 containing 100 μM H_2O_2. Samples (n=3) were incubated at 37℃ for certain time and an aliquot (100 μL) was withdrawn for each measurement.

Preparation and characterization of micelles. Micelles were prepared by dialysis method. In brief, 10 mg polymer and 1 mg curcumin were dissolved in 2 mL DMF. The solution was dialyzed against 10 mM PSB 7.4 for 24 hours with a membrane (MWCO: 3500) and further filtered with 0.22 μm membrane before use. The size distribution and PDI of prepared micelles were measured by DLS (Zetasizer Nano-ZS, Malvern 3600, Worcestershire, U.K.). The morphology of micelles was characterized by transmission electron microscopy (Tecnai G2 spirit Biotwin, FEI, Hillsboro). The loading efficiency of curcumin was measured by HPLC after extraction with methanol. HPLC method was set as: Agilent C18 column, 250mm, 1.0 mL/min, 58% acetonitrile + 42% 2% acetic acid, injection volume 10 μL, VWD wave length: 420 nm. The ROS scavenging ability of micelles were determined by measuring the remaining concentration

of H_2O_2 after incubation compared with an initial concentration of 10 μM with the Fluorometric Hydrogen Peroxide Assay Kit according to the manufacturer's instructions.

Measurement of Critical Micelle Concentration (CMC). Pyrene was used as a fluorescent probe to determine the CMC of APLB/CUR micelles. A 4 μL aliquot of pyrene stock solution (6×10^{-4} M in acetone) was added into empty vials and acetone was evaporated overnight. The vials were then filled with 400 μL of micelle solution at different concentrations and gently stirred overnight at room temperature to ensure pyrene incorporation into micelles. The maximum excitation of fluorescence at Em 337 nm was measured and plotted vs micelle concentration. Using linear regression, the equations describing the two linear parts of the plot were established and CMC was calculated from the intersection of these two lines.

Cell lines. Brain capillary endothelial cells (BCECs) were kindly provided by Prof. J.N. Lou (the Clinical Medicine Research Institute of the China-Japan Friendship Hospital). BCECs were expanded and maintained in special Dulbecco's modified Eagle medium (Sigma-Aldrich) supplemented with 20% heat-inactivated fetal bovine serum (FBS), 100 μg/mL epidermal cell growth factor, 20 μg/mL heparin, 2 mmol/L L-glutamine, 40 μU/mL insulin, 100 μg/mL streptomycin, and 100 U/mL penicillin. Human neuroblastoma SH-SY5Y cells were grown in F12/ Minimum Essential Media containing 100 U/mL penicillin, 100 mg/mL streptomycin, and 10% fetal bovine serum. Cells were incubated at 37 ℃ under a saturating humidity atmosphere containing 5% CO_2. Mouse microglia clone cells (Ra2) were kindly provided by Prof. Bin Ji (Molecular Imaging Center, National Institute of Radiological Sciences, Japan). Ra2 cells were grown in Eagle's MEM (Gibco) supplemented with 10% FBS, 0.2% glucose, 5 μg/mL bovine insulin and 1 ng/mL recombinant GM-CSF.

Investigation of cellular uptake and mechanisms of internalization. BCECs and SH-SY5Y cells were seeded in 24-well plates (Corning, New York) at a density of 1×10^4 cells/well and

incubated until 90% confluence was achieved. Cells were washed extensively and incubated with APLB/CUR with different Ab peptide modification (0%, 10%, 20%) at the concentration of 20 μg/mL curcumin for 30 min. Cells were then carefully washed and observed under a fluorescence microscope (Leica, Solms, Germany). To investigate the internalization mechanisms, BCECs and SH-SY5Y cells were seeded in 6-well plates at a density of 1×10^5 cells/well and incubated until 90% confluence was achieved. The cells were then preincubated under different inhibitive conditions with 10 mM PBS 7.4 containing Ab peptide (40 μg/mL, 20 equiv), filipin complex (2 μg/mL), colchicines (2 μg/mL), or phenylarine oxide (PhAsO, 1 μg/mL), respectively for 30 min, followed by addition of APLB/CUR (10 μg/mL curcumin) and incubation for 1 hour at 37℃ or 4℃. Then cells were washed extensively and collected to extract curcumin with ethyl acetate for HPLC quantification; untreated cells were also collected to exclude matrix effect. To investigate microglia uptake under Aβ activation, $A\beta_{25-35}$ was first prepared by dissolving in deionized water at 1 mM and aggregating at 37℃ for 10 days before use. Ra2 cells were seeded in 6-well plates at a density of 1×10^5 cells/well and incubated until 90% confluence was achieved. Cells were preincubated with $A\beta_{25-35}$ (20 μM) for 24 h and further incubated with PLB/CUR and APLB/CUR micelles for 1 hour. Curcumin was extracted as mentioned above and measured with HPLC.

Transcytosis across the in vitro BBB model. BCECs monolayer in a transwell system was established as the in vitro BBB model to investigate whether APLB/CUR could cross BBB. BCECs cells were seeded into a 0.3 cm^2 cell culture insert at a density of 2×10^4 cells/well and allowed to achieve confluence for several days. Coumarin-6 loaded micelles were prepared with the same procedure for APLB/CUR as described previously. 250 μL FBS-free medium containing different Cou6 loaded micelles was added to the donor chamber and 800 μL medium to the reception pool at time 0. ^{14}C-labeled sucrose was selected as an indicator and added along

with micelles to evaluate the permeability of the BBB model, which was also judged by TEER before and after the experiment. Cells were incubated at 37 ℃ on a rocking platform at 50 rpm while 50 μL samples were taken at 5, 10, 15 and 30 min for Cou6 and ^{14}C detection and 50 μL fresh medium was replenished. The apparent permeability (Papp) was calculated as described below.

Papp = (dQ/dt) * (1/C_0) * (1/A)

where dQ/dt is the permeability rate (nmol/s), C_0 is the initial concentration (nmol/ml) in the donor chamber, and A is the surface area (cm^2) of the membrane filter.

Intracellular ROS-responsive release study. SH-SY5Y cells were seeded in three confocal culture dishes (Corning, New York) at a density of 5000 cells/dish and incubated until 60% confluence was achieved. Coumarin loaded APLB micelles were prepared as mentioned above. All dished were treated with APLB/Cou6 for 30 min, and two of them were further treated with or without 100 μM H_2O_2 for another 60 min after wash. Hoechst 33342 (1 μg/mL, 20 min) and LysoTracker Deep Red (50 nM, 30 min) were applied to stain nuclei and late endosomes/lysosomes, respectively. After staining, all dishes were washed with PBS 7.4 and observed with the confocal fluorescence microscope by a 63× oil immersion lens (Carl Zeiss LSM710, Oberkochen, Germany).

Investigation of the neuroprotective effect of APLB/CUR micelles. Two different neurotoxic models were applied in this study. In the first model, SH-SY5Y cells were seeded in 96-well plate at a density of 5000 cells/well and incubated until 90% confluence was achieved. Cells were treated with CUR, PLB/CUR, APLB and APLB/CUR at a normalized CUR concentration of 10 μg/mL for 1 h. After extensive wash of the cells, H_2O_2 was added to the medium to a final concentration of 100 μM and was replenished every 4 h. Cells were incubated at 37℃ under this

condition for 12 h and MTT assay (n=6) was performed. Cells without H_2O_2 incubation served as control while cells without drug treatment served as blank group. In the second model, SH-SY5Y cells were seeded in 96-well plate at a density of 1×10^4 cells/well and incubated until 90% confluence was achieved. Cells were treated as mentioned above before the 24 h incubation of $A\beta_{25-35}$ (20 μM) and then carefully washed and further treated with DHE probe for 30 min. The solution was removed, and cells were washed and observed under a fluorescence microscope (Leica, Solms, Germany). For the apoptosis assay, cells were treated as mentioned above with drugs and $A\beta_{25-35}$, and then stained with Annexin V-FITC and propidium iodide for 15 min before observation under a fluorescence microscope (Leica, Solms, Germany).

Mice. C57BL/6 mice (male, 18~20 g) were purchased from the Sino-British SIPPR/BK Lab Animal Ltd. (Shanghai, China). APPswe/PSEN1dE9 mice (male, 18~20 g) were purchased from Nanjing Biomedical Research Institute of Nanjing University (Nanjing, China). All animal experiments were carried out in accordance with guidelines evaluated and approved by Fudan University Institutional Animal Care and Use Committee (IACUC).

Study of APLB/BODIPY distribution in APP/PS1 model mice. BODIPY (650/665) was encapsulated in APLB micelles as a NIR probe to track the biodistribution of micelles in AD mice by the preparation method mentioned above. Unmodified micelles PLB/BODIPY and modified micelles APLB/BODIPY were intravenously injected via tail vein with a normalized concentration of BODIPY to 6-month old APP/PS1 and WT mice. The mice were anesthetized and visualized under IVIS imaging system (Caliper) at 4 h post-injection, and then sacrificed to collect major organs and image ex vivo. Fluorescence intensity of BODIPY was quantified by IVIS. The brains were further dehydrated and fixed in 4% paraformaldehyde for 24 h before frozen section was performed. The sections were stained with DAPI to show nuclei and were immediately observed under a fluorescence microscope (Leica, Solms, Germany). Homogenized

organs of 3 mice in each group were mixed with methanol (1:4, v/v), vortexed for 2 min and centrifuged at 16000 rpm and 4 ℃ to collect the BODIPY-containing supernatant for HPLC measurement. The mobile phase for HPLC analysis was consisted of acetonitrile and 0.1% trifluoroacetic acid/water (70:30, v/v) at 1 mL/min. Fluorescence signal was monitored at 650 nm (Excitation) and 665 nm (Emission).

Treatment schedule. AD mice and wild type (WT) mice were maintained for about 9 months. 10 APP/PS1 mice and 10 WT mice were randomly grouped for immunofluorescence staining and western blotting at the 13th week and 25th week after birth before treatment (5 mice per group). At the 26th week after birth, 100 mice were randomly divided into five groups (20 mice per group) and intravenously injected with saline, curcumin, PLB/CUR, APLB/CUR (normalized curcumin dosage at 5 mg/kg) and APLB (equivalent amount of polymer to APLB/CUR) micelles, respectively once a week until the 37th week after birth. At the end of each month during treatment, five mice in each group were randomly picked and sacrificed for immunofluorescence staining and ELISA. At the 38th week after birth, the remaining mice (5 mice per group) were evaluated therapeutic effect by the Morris Water Maze test and sacrificed for immunofluorescence staining and ELISA.

Morris water maze test. The water temperature was maintained at 22 °C. The round pool was located in a soundproof test room with constant light. Four different geometric figures were placed on the wall of the pool which evenly divided the pool into four quadrants. A 10 cm diameter platform was placed in the center of quadrant 1 and its position was unchanged during the training trials. Water was filled in the pool until the surface was 1.5 higher than the platform, and titan white powder was added to make it invisible in the water. A digital camera was set above the pool and connected to a tracking device to record the path taken by the mice. Seven training trials per day were conducted for six consecutive days before the experimental test for

each group. Each mouse was released in the water facing wall of water maze at random starting position. The time to reach the platform was recorded in each trial with a maximal time limit of 60 s, and mouse was allowed to stay on the platform for 15 s. If the platform was not found within 60 s, the latency was recorded at 60 s and the mouse was gently guided to the platform and allowed to remain on the platform for 15 s. The time that the mouse spent in each quadrant was analyzed with a computerized tracking system.

Measurement of brain cytokines. Brain tissues were processed as described previously*[56]*. Protein concentration in the samples was determined with Bradford protein quantification kit according to the manufacturer's instructions. Samples from mice before treatment were analyzed by western blot. In brief, 40 μg total proteins were separated by 12% SDS-PAGE and transferred onto PVDF membranes at 300 mA for 2 h (Mini-PEOTEAN, Bio-Rad). The membranes were blocked with 5% BSA for 1 h and probed with primary antibodies (TNF-α, IL-1β, β-actin) at 4℃ overnight, followed by rinse and incubation with horseradish peroxidase-conjugated secondary antibody at room temperature for 1 h. Bands were visualized by ECL detection reagents and analyzed by Image Lab (Bio-Rad) and normalized by β-actin. TNF-α and IL-1β in the samples from mice during treatment were analyzed by ELISA according to the manufacturer's instructions.

Immunofluorescence staining. Mice were sacrificed as scheduled to perform immunostaining assays. In brief, mice were anaesthetized and perfused transcardially with saline and 4% paraformaldehyde. Brains were immediately removed and further fixed for 24 h, followed by dehydration in 15% sucrose and 30% sucrose at 4℃. Frozen coronal sections of 20 μm in thickness were prepared and processed for immunofluorescence staining using primary antibodies: RAGE antibody (1:1000), 8OHG antibody (1:1000), Aβ (82E1) antibody (1:1000), Iba-1 antibody (1:1000) and NeuN antibody (1:1000). Secondary antibodies including Goat anti-mouse

IgG Alexa Flour 488, Goat anti-rabbit IgG Alexa Flour 488 and Goat anti-rabbit IgG Alexa Flour 549 were applied for fluorescence microscope imaging.

Immunohistological staining study. Major organs (heart, liver, spleen, lung, and kidney) of mice with 3-month treatment were removed along with brains and fixed in 4% paraformaldehyde for 24 h at 4℃. Organs were embedded in paraffin and sections of 5 μm were prepared for hematoxylin and eosin (H&E) staining.

Dot Blot Assay. Dot blot assay was performed using oligomer specific (antioligomer A11, Intrivogen) antibody. For in vitro inhibition assay, $A\beta_{25\text{-}35}$(100 μM) were pretreated with hexafluoroisopropanol overnight and diluted with DI water for further use. $A\beta_{25\text{-}35}$ monomers were mixed with different formulations (curcumin was normalized to 10 μg/mL if contained). Samples were adjusted to pH 3 and incubated at 42℃ for 2.5 h, followed by 48 h stirring at room temperature before dot blot assay was performed. In vitro samples (3 μL) or brain samples (5μL, 20 μg) were blotted onto nitrocellulose membrane and air-dried. After 1 h blocking in 5% milk containing tris-buffered saline (TBS) with 0.2% (v/v) tween (TBS-T) at room temperature, the membranes were incubated for 2 hours at room temperature with primary antibodies (A11, 1:500 dilution). The membranes were then washed three times with TBS-T and incubated with HRP-conjugated secondary anti-rabbit antibody for 1 h at room temperature. After five washes, the antigen and antibody binding were detected using the Meilunbio FG Super Sensitive ECL Luminsecence Reagent (Meilun, China).

Real-Time Quantitative RT-PCR Analysis. For quantitative evaluation of RNA levels of associated proteins, total RNA from hippocampus and cortex in mice brain was isolated using Trizol LS reagent (Invitrogen, USA) according to the manufacturer's instructions. The RT-PCR analysis was performed by Wuhan Servicebio Co. Ltd. Primer sequence: GSK-3β: 5' TCCGAGGAGAGCCCAATGTT; GSK-3β AS: 5' GGACCTTTATTATTTCCACCAACTG;

APP: 5’ CCAAGAGGTCTACCCTGAACTGC; APP AS: 5’ AGGCAACGGTAAGGAATCACG; PS1: 5’ TGGCTCATCTTGGCTGTGATT; PS1 AS: 5’ ACTGAAGCCACCATCATCGTT; iNOS: 5’ GGGCTGTCACGGAGATCAATG; iNOS AS: 5’ GCCCGGTACTCATTCTGCATG; GAPDH: 5’ AGGAGCGAGACCCCACTAACA; GAPDH AS: 5’ AGGGGGGCTAAGCAGTTGGT.

Statistical analysis. Results were analyzed by GraphPad Prism software and presented as mean ± SD. The statistical significance was evaluated by student’s t-test and one-way ANOVA. P value <0.05 was considered statistically significant.
